# CD37 regulates the self-renewal of leukemic stem cells via integrin-mediated signaling in acute myeloid leukemia

**DOI:** 10.1016/j.stemcr.2025.102573

**Published:** 2025-06-28

**Authors:** Jinyuan Lu, Lixin Lv, Xiaoxue Tian, Zheng Li, Yuting Ma, Nannan Li, Jian Wang, Guangming Wang, Yu Zeng, Wenjun Zhang, Jun Xu, Aibin Liang

## Main text

(Stem Cell Reports *20*, 102476; May 13, 2025)

In the originally published version of this article, only Aibin Liang was listed as a corresponding author, and Wenjun Zhang and Jun Xu mistakenly were not, although the nature of their contributions should have given all three authors this status. Additionally, the contributions of Jinyuan Lu and Lixin Lv were not equal, but they shared an equal contributions footnote in the originally published article. After discussion among and approval from all authors, the author list has been corrected online. The authors apologize for any inconvenience caused.

