# Supplementary material for: CD37 regulates the self-renewal of leukemic stem cells via integrin-mediated signaling in acute myeloid leukemia
Source: Stem Cell Reports. 2025 Apr 17;20(5):102476. doi: 10.1016/j.stemcr.2025.102476 (PMC12143136; doi:10.1016/j.stemcr.2025.102476)
Supplement: Document S1. Figures S1–S7, Tables S1–S3, and supplemental methods [file mmc1.pdf]

Stem Cell Reports, Volume *20*

Supplemental Information

CD37 regulates the self-renewal of leukemic stem cells via integrin-mediated signaling in acute myeloid leukemia

Jinyuan Lu, Lixin Lv, Xiaoxue Tian, Zheng Li, Yuting Ma, Nannan Li, Jian Wang, Guangming Wang, Yu Zeng, Wenjun Zhang, Jun Xu, and Aibin Liang

Supplemental Information

Supplemental Figures

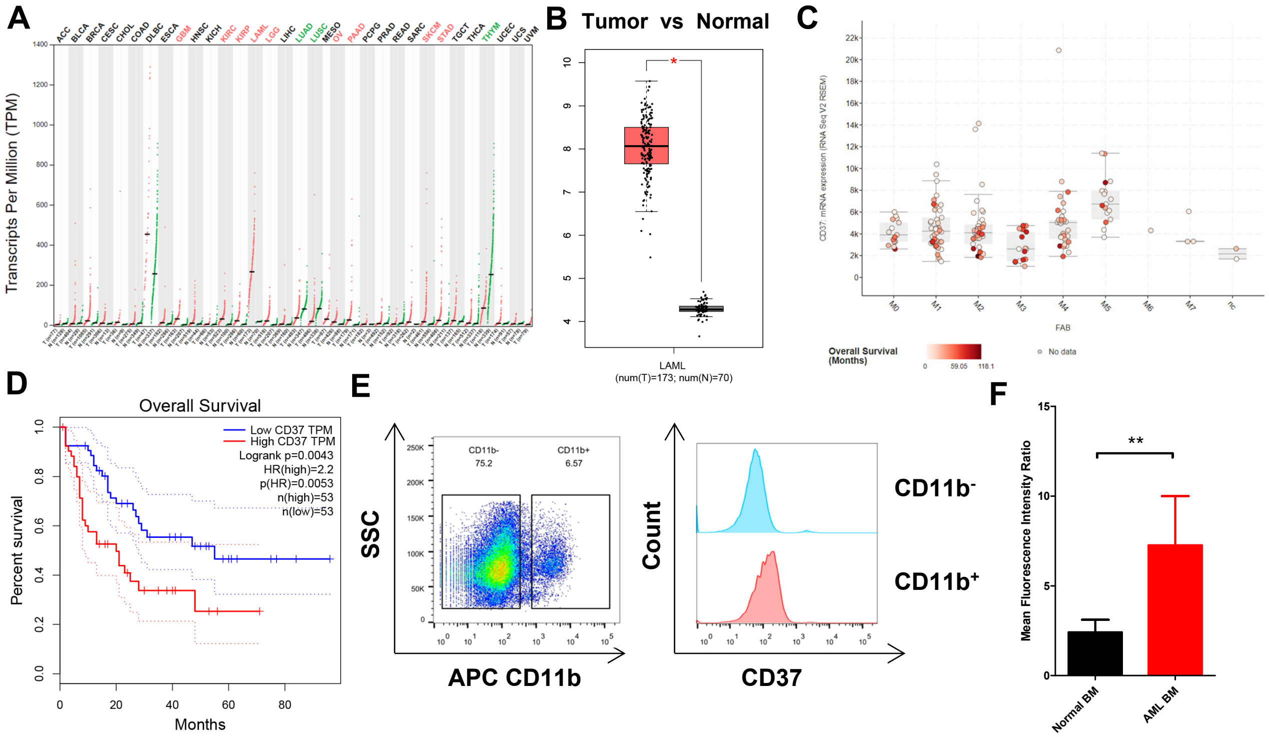

Figure S1. CD37 was upregulated in human AML cells

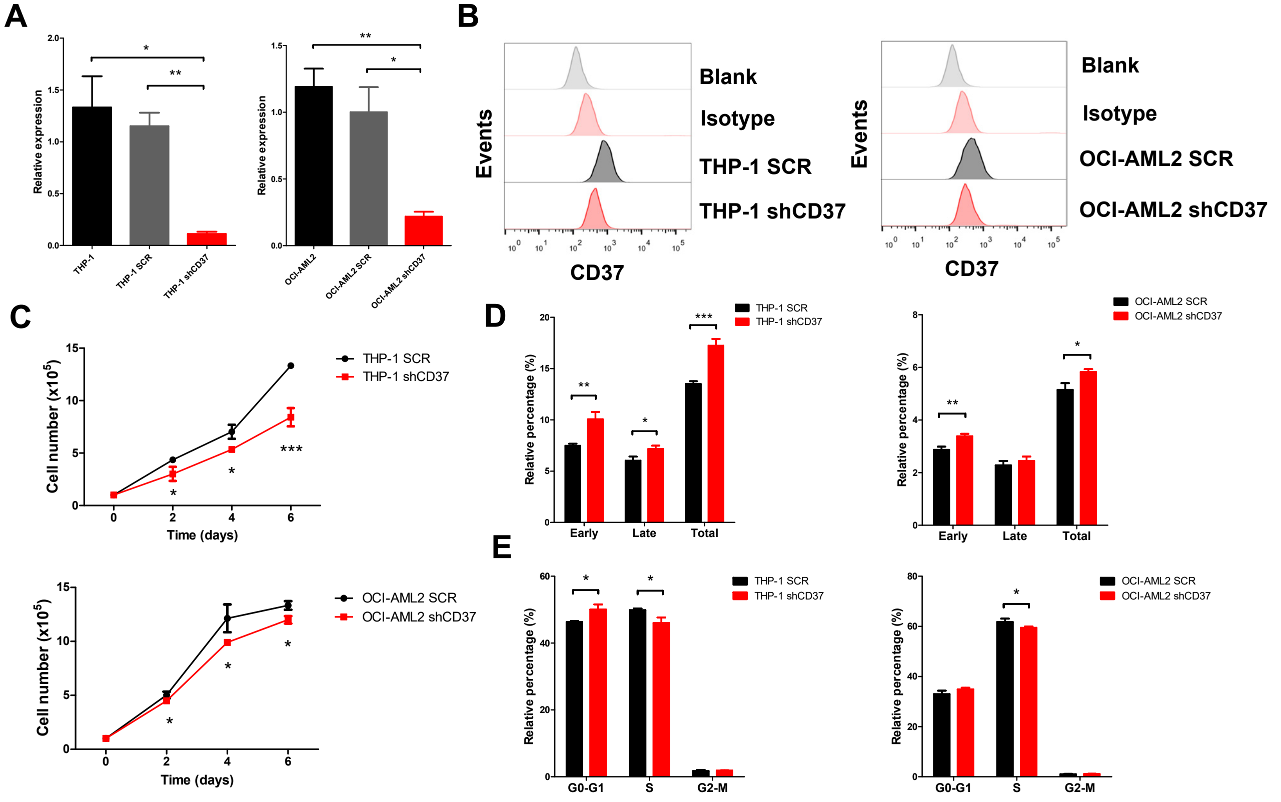

Figure S2. CD37 knockdown inhibited the proliferation of human AML cell lines

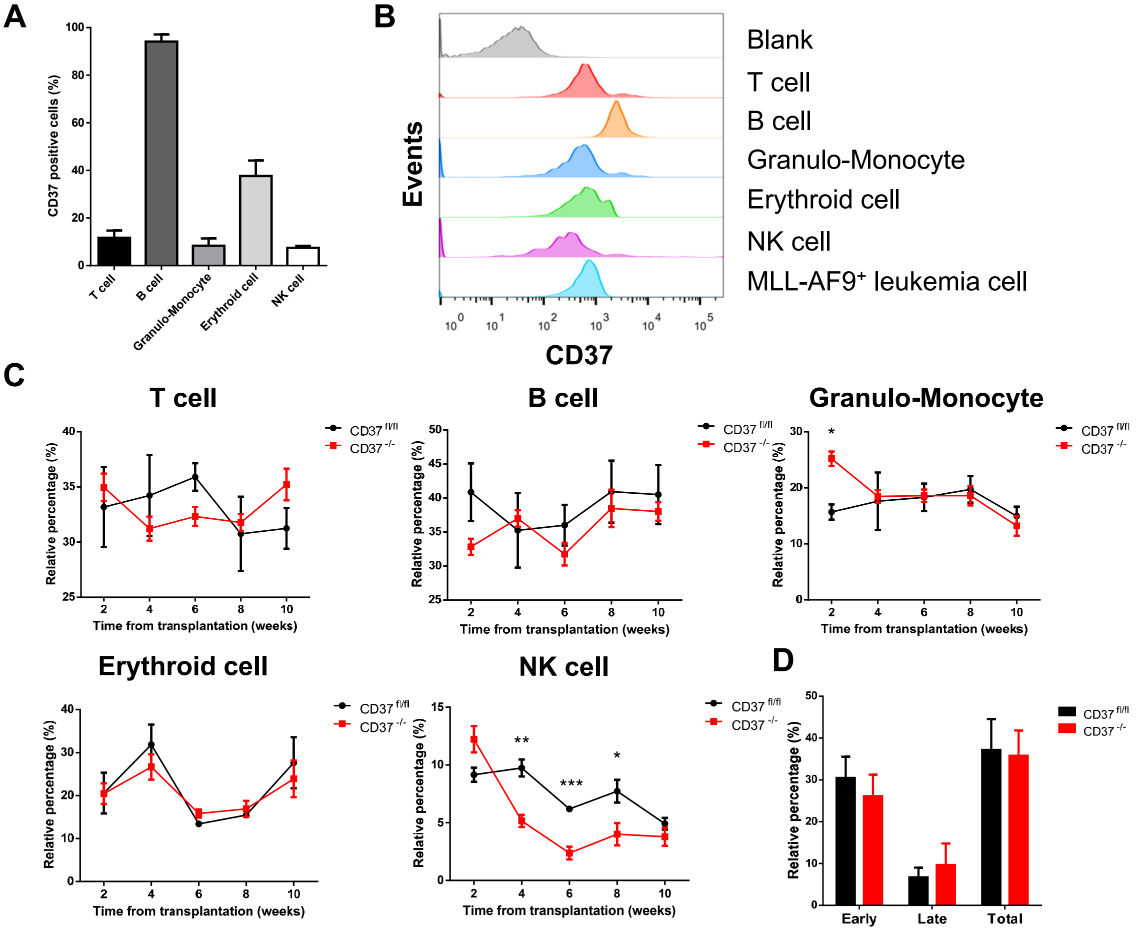

Figure S3. CD37 deficiency had a minor effect on normal BM cell differentiation

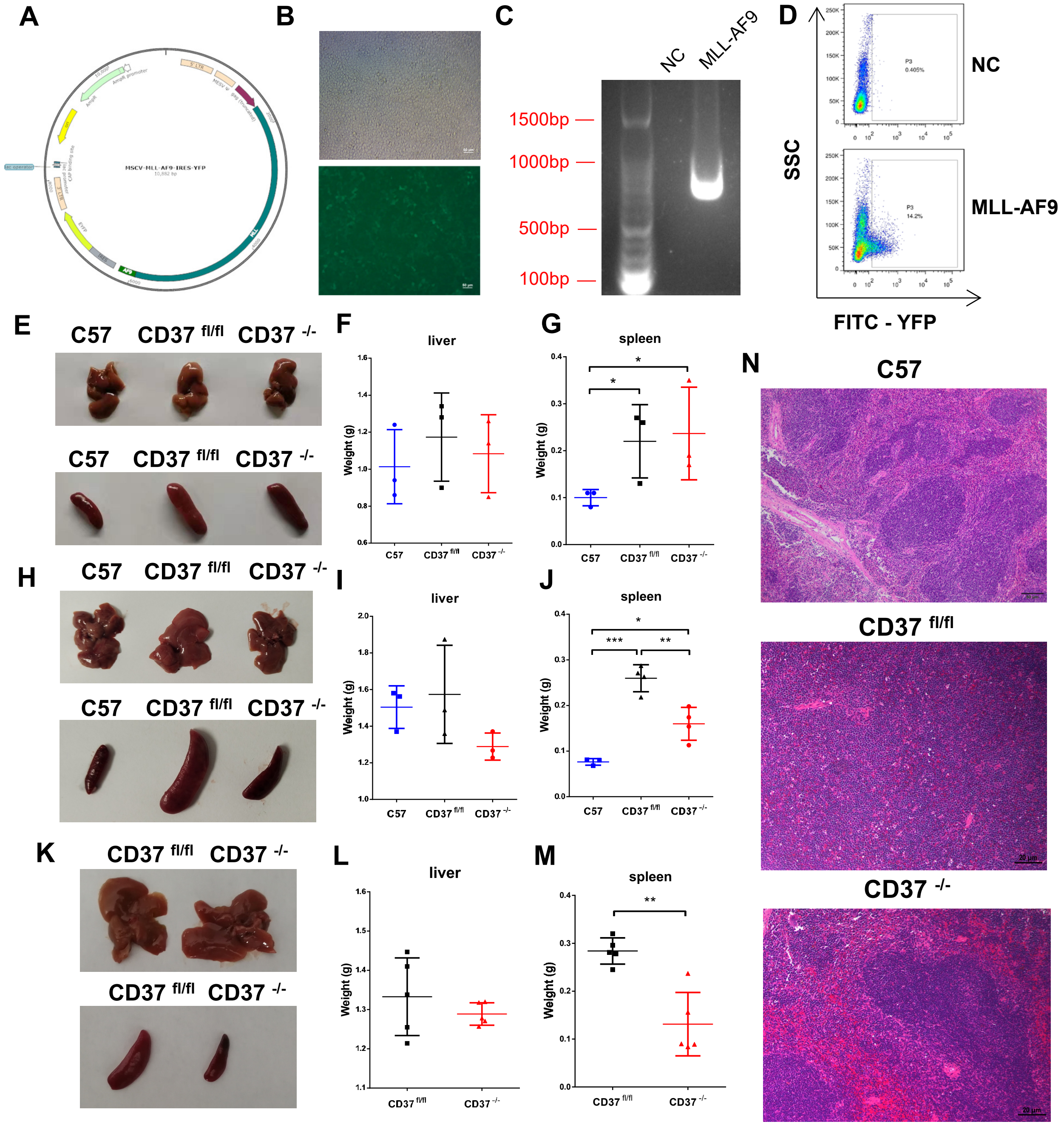

Figure S4. MLL-AF9 transfection efficacy and pathological changes in MLL-AF9 AML

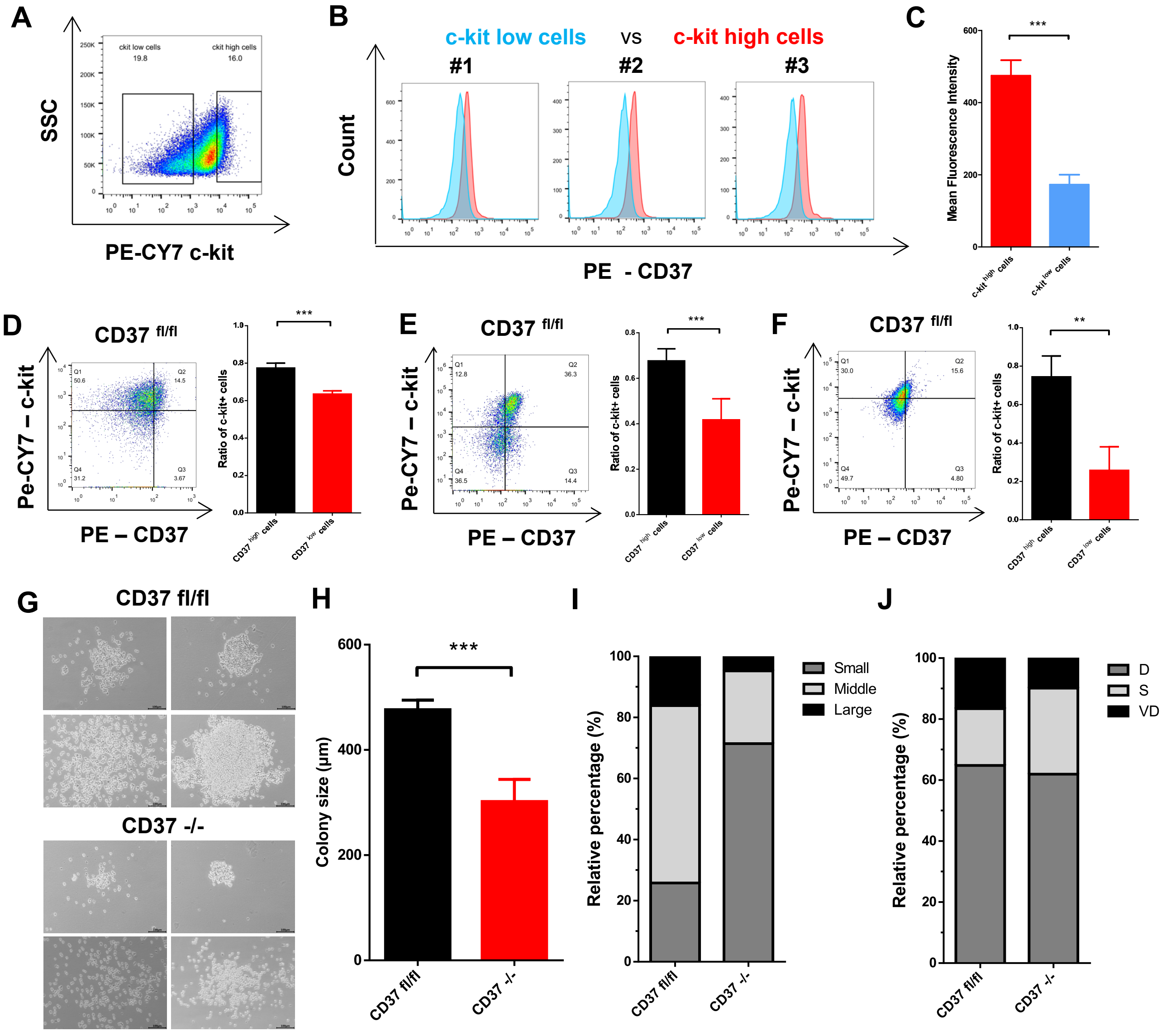

Figure S5. CD37 was enriched in LSCs and CD37 deficiency impaired the colony formation of LSCs

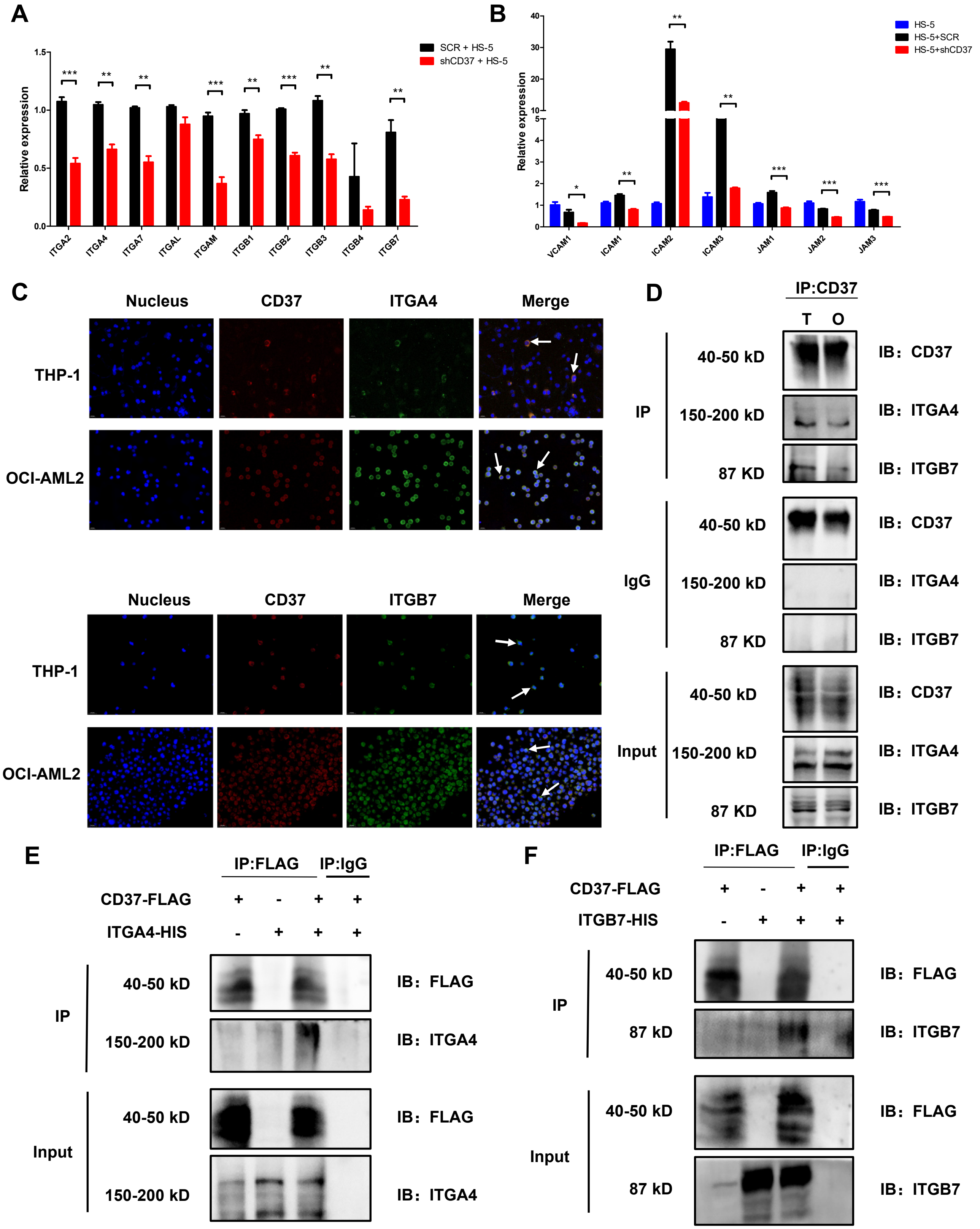

Figure S6. CD37 regulated the expression of integrins and interacted with integrin α4β7

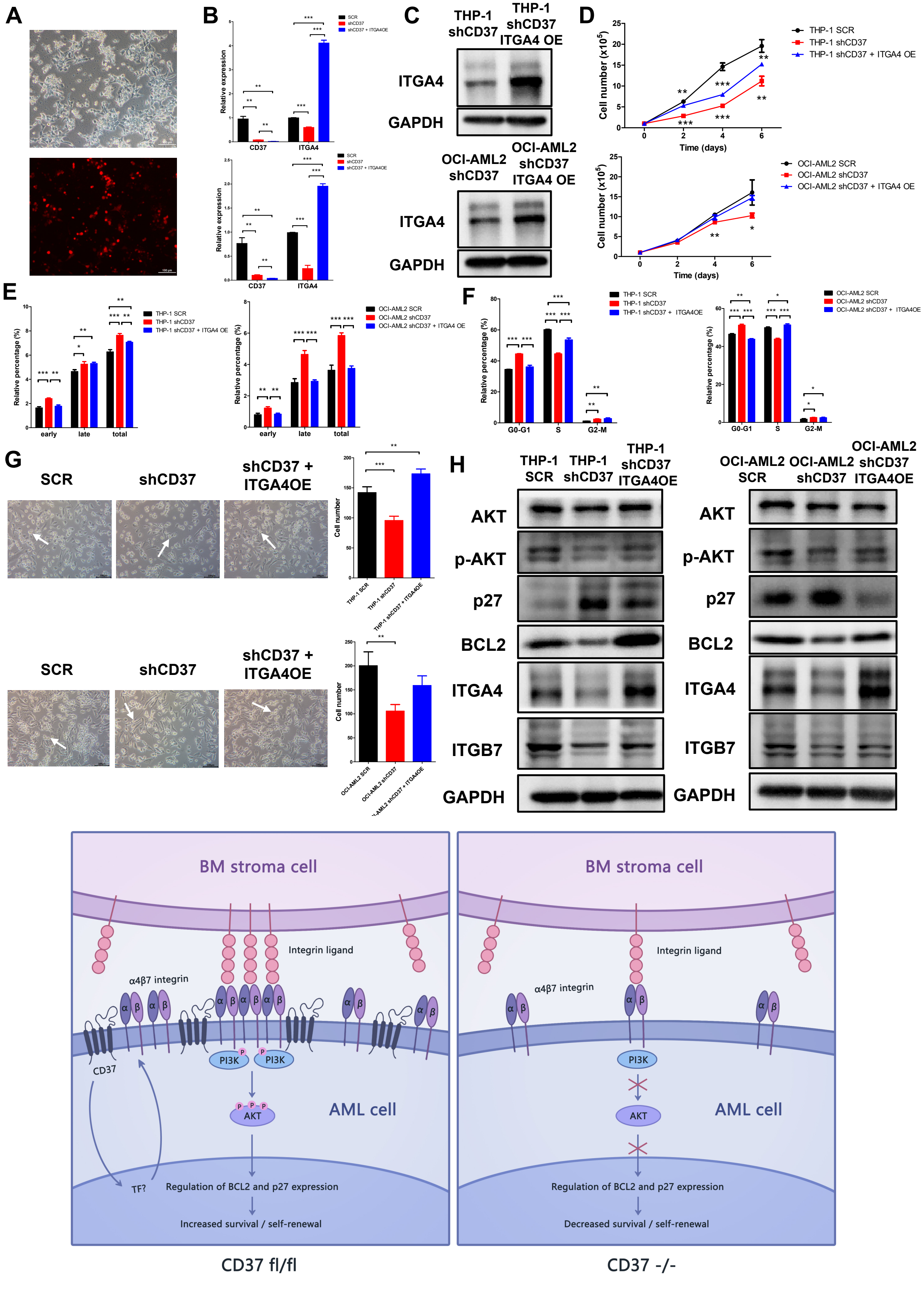

Figure S7. ITGA4 overexpression rescued the phenotypic effects caused by CD37 loss

Supplemental figure legends

Figure S1. CD37 was upregulated in human AML cells.

(A-B) CD37 expression profile among all TCGA tumors (A) or specifically in LAML (B). The data were analyzed using the online database GEPIA.

(C) CD37 expression in different AML subtypes. Dot color represents the duration of overall survival (OS), A deeper red color suggests a longer OS, whereas a lighter white color indicates a shorter OS. The image was depicted on cBioPortal.

(D) Survival plot for LAML patients with high or low expression of CD37. The survival data were analyzed using the online database GEPIA.

(E) Representative flow cytometric analysis of CD37 expression in $CD11b^-$ or $CD11b^+$ AML cells.

(F) The mean fluorescence intensity ratio (MFIR) of CD37 in $CD11b^+$ normal and AML BM cells (n=4 for normal BM and n=7 for AML BM). Error bars in (F) were defined as mean ± SD. $*p < 0.05$, $**p < 0.01$.

Figure S2. CD37 knockdown inhibited the proliferation of human AML cell lines.

(A-B) qPCR and flow cytometric validation of CD37 knockdown efficiency in THP-1 and OCI-AML2 cells with another sequence targeting CD37 (shCD37-2).

(C) Cell proliferation in THP-1 and OCI-AML2 cells transfected with SCR or shCD37.

(D) Quantification of early, late and total apoptotic cells in THP-1 and OCI-AML2 cells transfected with SCR or shCD37.

(E) Quantification of cells in G0-G1 phase, S phase or G2-M phase in THP-1 and OCI-AML2 cells transfected with SCR or shCD37. Error bars in (A), (C), (D) and (E) were defined as mean ± SD. *$p < 0.05$, **$p < 0.01$, ***$p < 0.001$.

Figure S3. CD37 deficiency had a minor effect on normal BM cell differentiation.

(A) CD37 positive rate in murine T cells, B cells, granulo-monocytes, erythroid cells and NK cells (n=4).

(B) Flow cytometric validation of CD37 expression in different types of murine cells.

(C) The trends in the proportions of T cells, B cells, granulo-monocytes, erythroid cells and NK cells in the peripheral blood following tamoxifen or corn oil administration (n=4 for each).

(D) Quantification of apoptotic NK cells in the peripheral blood of CD37 $^{fl/fl}$ and CD37 $^{-/-}$ mice at week 10 (n=4 for each). Error bars in (A), (C) and (D) were defined as mean ± SD. *$p < 0.05$, **$p < 0.01$, ***$p < 0.001$.

Figure S4. MLL-AF9 transfection efficacy and pathological changes in MLL-AF9 AML.

(A) The structure of MSCV-MLL-AF9-IRES-YFP plasmid.

(B) Fluorescence imaging of YFP^+ cells (scale bar = 50μm).

(C) PCR validation of MLL-AF9 expression in Lin^- cells transfected with MSCV-MLL-AF9-IRES-YFP. The PCR primers targeting MLL-AF9 were listed in Supplementary Table 2.

(D) Flow cytometric validation of YFP expression in Lin^- cells transfected with MSCV-MLL-AF9-IRES-YFP.

(E, H and K) The morphology of livers and spleens obtained from normal C57 mice or C57 mice transplanted with $CD37^{fl/fl}$ or $CD37^{-/-}$ AML LSCs. (E) primary AML; (H) secondary AML; (K) tertiary AML.

(F-G, I-J and L-M) Liver and spleen weight of normal C57 mice or C57 mice transplanted with $CD37^{fl/fl}$ or $CD37^{-/-}$ AML LSCs. (F-G) primary AML (n=3); (I-J) secondary AML (n=4); (L-M) tertiary AML (n=5).

(N) Hematoxylin-Eosin (HE) staining for the spleen tissues obtained from normal C57 mice (upper panel, scale bar = 50μm) or C57 mice transplanted with $CD37^{fl/fl}$ or $CD37^{-/-}$ AML LSCs (middle and lower panel, respectively, scale bar = 20μm) in the secondary AML. Error bars in (F), (G), (I), (J), (L) and (M) were defined as mean ± SD. *$p < 0.05$, **$p < 0.01$, ***$p < 0.001$.

Figure S5. CD37 was enriched in LSCs and CD37 deficiency impaired

the colony formation of LSCs.

(A-C) Representative flow cytometric analysis of CD37 expression in c-kit ^{high} or c-kit ^{low} AML LSCs (A-B). The MFI of CD37 in c-kit ^{high} or c-kit ^{low} AML LSCs was also quantified (C, n=3).

(D-F) Representative flow cytometric analysis of CD37 and c-kit expression in CD37$^{fl/fl}$ AML LSCs. The proportion of c-kit^{+} cells in CD37 ^{high} cells or CD37 ^{low} cells was also quantified. (D) primary AML (n=4); (E) secondary AML (n=5); (F) tertiary AML (n=3).

(G) Representative images demonstrating the colony formation of CD37 $^{fl/fl}$ and CD37 $^{-/-}$ AML LSCs in the secondary transplantation model (scale bar = 100μm).

(H) Quantification of colony size for each group (indicated as diameter, μm).

(I) Quantification of small (<370μm), middle (370-630μm) and large (>630μm) colonies for each group. By depicting a histogram to represent colony sizes, optimal intervals for the cutoff values were determined.

(J) Quantification of dense (D), very dense (VD) and sparse (S) colonies for each group. Error bars in (C), (D), (E), (F) and (H) were defined as mean ± SD. $**p < 0.01$, $***p < 0.001$.

Figure S6. CD37 regulated the expression of integrins and interacted with integrin α4β7.

(A-B) qPCR analysis for the expression of integrins on OCI-AML2 cells (A) and integrin ligands on HS-5 stroma cells (B). OCI-AML2 cells transfected with SCR or shCD37 were co-cultured with HS-5 cells for 24 hours and subjected to RNA extraction, cDNA synthesis and qPCR verification.

(C) Representative immunofluorescence images (40x, scale bar = 20μm) demonstrating the location of CD37 and ITGA4/ITGB7 on the membrane of THP-1 and OCI-AML2 cells. The white arrows indicate co-localization of CD37 and ITGA4/ITGB7.

(D) Endogenous co-immunoprecipitation assay for THP-1 (T) and OCI-AML2 (O). CD37 antibody was applied for immunoprecipitation. Simultaneously, rabbit IgG was employed as negative control. A portion of the protein lysates were utilized as input samples. Antibodies targeting CD37, ITGA4 and ITGB7 were administrated for subsequent immunoblotting. The 40-50KD band immunoprecipitated by CD37 antibody in the IgG group represent nonspecific binding of Fc region of the antibody.

(E-F) Exogenous co-immunoprecipitation assay. (E) 293T cells were transfected with CD37-FLAG, ITGA4-HIS or both, and subjected to co-IP and immunoblotting. (F) 293T cells were transfected with CD37-FLAG, ITGB7-HIS or both, and subjected to co-IP and immunoblotting. Error bars in (A) and (B) were defined as mean ± SD. *$p < 0.05$, **$p < 0.01$, ***$p <$

0.001.

Figure S7. ITGA4 overexpression rescued the phenotypic effects caused by CD37 loss.

(A-C) Fluorescence imaging, qPCR and Western blot validation of ITGA4 overexpression. (A) Scale bar = 100μm.

(D) Cell proliferation in THP-1 and OCI-AML2 cells transfected with SCR, shCD37 or shCD37 + ITGA4 OE.

(E-F) Quantification for apoptosis (E) and cell cycle distribution (F) in THP-1 and OCI-AML2 cells transfected with SCR, shCD37 or shCD37 + ITGA4 OE.

(G) Representative images demonstrating the adherence of THP-1 cells (upper, scale bar = 100μm) or OCI-AML2 cells (lower, scale bar = 100μm) to HS-5 stroma cells. The white arrows indicate adherent AML cells. The number of adherent AML cells was also quantified.

(H) Immunoblotting for key DEGs in THP-1 and OCI-AML2 cells transfected with SCR, shCD37 or shCD37 + ITGA4 OE.

(I) The schematic diagram illustrating the regulatory role of CD37 in AML. CD37 facilitates the transduction of integrin mediated PI3K-AKT signaling in AML cell lines as well as AML LSCs. In the absence of CD37, integrins (e.g. $\alpha4\beta7$) undergo transcriptional downregulation and reduced accumulation, followed by diminished AKT phosphorylation, decreased

BCL2 expression and elevated p27 expression, which collectively contribute to a compromised AML cell survival. Error bars in (B), (D), (E), (F) and (G) were defined as mean ± SD. *$p < 0.05$, **$p < 0.01$, ***$p < 0.001$.

Supplementary Table 1

qPCR primers

Name	Sequence
muCd37-F	TTTGTCCTTCGTGCCACTG
muCd37-R	AATGAGGATGCCCAGGGTAAT
muCcnd2-F	GAGTGGGAACTGGTAGTGTTG
muCcnd2-R	GCACAGAGCGATGAAGGTC
muCdkn1b-F	TCAAACGTGAGAGTGTCTAACG
muCdkn1b-R	CCGGGCCGAAGAGATTTCTG
muBcl2-F	GCTACCGTCGTGACTTCGC
muBcl2-R	CCCCACCGAACTCAAAGAAGG
muItgb7-F	AAACGGTGCTGCCCTTTGTAA
muItgb7-R	CTCTCTCTCGAAGGCTTGAGC
muPik3cb-F	CTATGGCAGACAACCTTGACAT
muPik3cb-R	CTTCCCGAGGTACTTCCAACT
muGapdh-F	TGACCTCAACTACATGGTCTACA
muGapdh-R	CTTCCCATTCTCGGCCTTG
huCD37-F	CCAACGACTCCACAATCCTAGA
huCD37-R	GGCAAATGCCCACTATGGAAAT
huITGA2-F	CCTACAATGTTGGTCTCCCAGA
huITGA2-R	AGTAACCAGTTGCCTTTTGGATT
huITGA4-F	AGCCCTAATGGAGAACCTTGT

huITGA4-R	CCAGTGGGGAGCTTATTTTCAT
huITGA7-F	CTGACTCCATGTTCGGGATCA
huITGA7-R	CACCTGTGAAGGTTTGGCG
huITGAL-F	TGCTTATCATCATCACGGATGG
huITGAL-R	CTCTCCTTGGTCTGAAAATGCT
huITGAM-F	GCCTTGACCTTATGTCATGGG
huITGAM-R	CCTGTGCTGTAGTCGCACT
huITGB1-F	CAAGAGAGCTGAAGACTATCCCA
huITGB1-R	TGAAGTCCGAAGTAATCCTCCT
huITGB2-F	AAGTGACGCTTTACCTGCGAC
huITGB2-R	AAGCATGGAGTAGGAGAGGTC
huITGB3-F	CATGAAGGATGATCTGTGGAGC
huITGB3-R	AATCCGCAGGTTACTGGTGAG
huITGB4-F	GCTTCACACCTATTTCCCTGTC
huITGB4-R	GACCCAGTCCTCGTCTTCTG
huITGB7-F	GCGCATTGGTTTTGGTTCCT
huITGB7-R	AGATTGCCGGACACACTCTG
huVCAM1-F	GGGAAGATGGTCGTGATCCTT
huVCAM1-R	TCTGGGGTGGTCTCGATTTTA
huICAM1-F	ATGCCCAGACATCTGTGTCC
huICAM1-R	GGGGTCTCTATGCCCAACAA
huICAM2-F	CGGATGAGAAGGTATTCGAGGT

huICAM2-R	CACCCACTTCAGGCTGGTTAC
huICAM3-F	GGAGTTCCTTTTGCGGGTG
huICAM3-R	TCAGAGCTGGGACAATCAGTA
huJAM1-F	GTGCCTACTCGGGCTTTTCTT
huJAM1-R	GTCACCCGGTCCTCATAGGAA
huJAM2-F	GCAGTAGAGTACCAAGAGGCT
huJAM2-R	AGACACTCCGACCCAGTTTCT
huJAM3-F	CGGCTGCCTGACTTCTTCC
huJAM3-R	TGGGGTTCGATTGCTGGATTT
huGAPDH-F	CTGGGCTACACTGAGCACC
huGAPDH-R	AAGTGGTCGTTGAGGGCAATG

Supplementary Table 2

PCR primers

Name	Sequence
CD37-F	GGTTACCCCTGGCTACTGA
CD37-R	ATCCCCCTGTCTCTATCTTGA
Cre-F	GAACCTGATGGACATGTTCAGG
Cre-R	AGTGCGTTCGAACGCTAGAGCCTGT
MLL-AF9-F	CGTCGAGGAAAAGAGTGA
MLL-AF9-R	ATGTTTCCAGGTAACTCTGTAGT
Mycoplasma-F	GGGAGCAAACAGGATTAGATACCCT
Mycoplasma-R	TGCACCATCTGTCACTCTGTTAACCTC

Supplementary Table 3

Human patient information

ID	Gender	Age	Source	Diagnosis
1	Male	68	Bone marrow	AML-M4
2	Female	74	Bone marrow	AML-M1
3	Male	58	Bone marrow	AML-M5
4	Male	34	Bone marrow	AML-M4
5	Female	51	Bone marrow	AML-M5
6	Male	35	Bone marrow	AML-M4
7	Female	75	Bone marrow	AML-M5

Supplemental Methods

Cell lines, animals and patient samples

THP-1, OCI-AML2, MV4-11, K562, NALM-6, JURKAT, HEL, SUP-B15, HL-60, KG-1α, NB4, 293T and OP9 cells were purchased from the Cell Bank of Shanghai Institutes for Biological Sciences. HS-5 was purchased from Shanghai Fuheng Biotechnology Co. Ltd.

CD37 conditional knockout mice (CD37$^{fl/+}$) were established by Shanghai OBiO Technology Co. Ltd using the CRISPR-Cas9 system. *LoxP* alleles were introduced upstream of exon 4 and downstream of exon 5 within the *Cd37* genome. Rosa26-CreERT2 mice were purchased from Nanjing Biomedical Research Institute of Nanjing University. C57 BL/6 J mice (male, 6-8 weeks) were purchased from Shanghai Bikai Keyi Biotechnology Co. Ltd. CD45.1 mice were acquired from Professor Caiwen Duan from Shanghai Jiaotong University School of Medicine as a kind gift. For the generation of CD37$^{fl/fl}$, Cre^{+} mice, Mature CD37$^{fl/+}$ mice (F_0) were self-crossed to obtain homozygous CD37$^{fl/fl}$ mice (F_1). Next, CD37$^{fl/fl}$ mice and Rosa26-CreERT2 mice were crossed to obtain CD37$^{fl/fl}$, Cre^{+} mice (F_2, F_3, …). The presence of *LoxP* alleles and *Cre* sequences were confirmed by PCR. All mice were kept in specific pathogen-free facilities at the Animal Experiment Center of Tongji University. All animal experiments were conducted in accordance with the ethical approval from

the Animal Ethics Committee of Tongji University.

AML BM samples were obtained from the Hematology Department of Shanghai Tongji Hospital with approval from the Ethical Review Committee of Biomedical Research of Shanghai Tongji Hospital. Informed consent was obtained from all subjects. The clinical information of AML patients was listed in Supplementary Table 3. AML cells were cultured in RPMI 1640 medium with 20% FBS, 1% PS, 10 ng/ml human IL-3, IL-6, SCF, G-CSF and FLT3LG.

ShRNA sequences

The shRNA sequences targeting CD37 was named as shCD37 in this study. A scrambled sequence named SCR was employed as a control sequence to remove off-target effects induced by shRNA. The sequences were as follows:

SCR-F:

GATCCGTGCGCGCTTTGTAGGATTCGTTTCAAGAGAACGAATCC

TACAAAGCGCGCATTTTTG

SCR-R:

AATTCAAAAATGCGCGCTTTGTAGGATTCGTTCTCTTGAAACG

AATCCTACAAAGCGCGCACG

shCD37-1F:

GATCCGCGACTCCACAATCCTAGATAATTCAAGAGATTATCTAG

GATTGTGGAGTCGTTTTTG

shCD37-1R:

AATTCAAAAACGACTCCACAATCCTAGATAATCTCTTGAATTAT

CTAGGATTGTGGAGTCGCG

shCD37-2F:

GATCCGCTCGATATTCCTGTGCAGAAATTCAAGAGATTTCTGCA

CAGGAATATCGAGTTTTTG

shCD37-2R:

AATTCAAAAACTCGATATTCCTGTGCAGAAATCTCTTGAATTT

CTGCACAGGAATATCGAGCG

Cell apoptosis

Cells were collected and resuspended in 1x Binding Buffer. Annexin V-Alexa Fluor 647 and PI Staining Solution (YEASEN, 40304) were added to the cell suspension, followed by incubation at room temperature for 15min under dark conditions. The cell suspension was then strained with a 70μm filter and subjected to further analysis via flow cytometry. Where indicated, AML cells co-cultured with BM stroma cells were treated with Ara-C (1mM, 1:1000) and incubated at 37°C for 24h. Subsequently, the AML cells were washed 3-4 times with PBS and subjected to cell apoptosis assay.

Cell cycle analysis

Cells were incubated with BrdU (10mg/ml, 1:1000) at 37°C for 2h, and fixed with 75% ethanol at -20°C overnight. The cells were washed with staining buffer (PBS + 1% FBS + 0.09% NaN_3) and resuspended in permeabilization buffer (2N HCl + 0.05% Triton-X100 + ddH_2O) at room temperature for 30min. Subsequently, the samples were washed with staining buffer and resuspended in 0.1M $Na_2B_4O_7$ at room temperature for 2 min. After resuspension in staining buffer, the cells were incubated with APC BrdU antibody (Invitrogen, 17-5071-42) for 30min, and with PI/RNase staining buffer (BD, 550825) for 15min. The cell suspension was then strained with a 70μm filter and subjected to further analysis via flow cytometry.

Cell differentiation assay

The hematopoietic stem cells, progenitors and mature cells were labeled with different antibodies summarized as follows:

LT-HSCs: Lin^-, $c\text{-}kit^+$, $SCA\text{-}1^+$, $FLK2^-$, $CD34^-$; ST-HSCs: Lin^-, $c\text{-}kit^+$, $SCA\text{-}1^+$, $FLK2^-$, $CD34^+$; MPPs: Lin^-, $c\text{-}kit^+$, $SCA\text{-}1^+$, $FLK2^+$, $CD34^+$; CMPs: Lin^-, $c\text{-}kit^+$, $SCA\text{-}1^-$, $CD16/32^{low}$, $CD34^+$, $IL\text{-}7R^-$; MEPs: Lin^-, $c\text{-}kit^+$, $SCA\text{-}1^-$, $CD16/32^-$, $CD34^-$, $IL\text{-}7R^-$; GMPs: Lin^-, $c\text{-}kit^+$, $SCA\text{-}1^-$, $CD16/32^+$, $CD34^+$, $IL\text{-}7R^-$; CLPs: Lin^-, $c\text{-}kit^{low}$, $SCA\text{-}1^{low}$, $IL\text{-}7R^+$; T cells: $CD3^+$; B cells: $CD19^+$; Granulo-Monocytes: $MAC\text{-}1^+$, $GR\text{-}1^+$; Erythroid

cells: $TER119^+$; NK cells: $NK1.1^+$.

Cell adhesion assay

$5x10^5$ YFP^+ AML cells were plated on OP9 cells cultured in MEM-α with 20% FBS, 1% PS, 10 ng/ml IL-3, 10 ng/ml IL-6, 10 ng/ml SCF and 10 ng/ml G-CSF in a 12-well plate, and incubated at 37°C for 24h. The wells were washed 3-4 times with PBS in order to remove unattached cells. Images were captured under an inverted microscope and the adherent AML cells were quantified in Image J. For adhesion assay of AML cell lines, $5x10^5$ AML cells were plated on HS-5 cells cultured in DMEM with 10% FBS and 1% PS in a 12-well plate, and the subsequent procedures were consistent with the aforementioned steps.

Transcriptome analysis

$2x10^6$ YFP^+, c-kit^+ cells isolated from leukemia mice transplanted with $CD37^{fl/fl}$ or $CD37^{-/-}$ AML LSCs were sorted by flow cytometry and lysed with trizol reagent (Invitrogen, 15596026). The samples were sent to Shanghai Hongxu Biotechnology Co. Ltd and sequenced with Illumina Novaseq™ 6000. The downstream analysis was accomplished in R 4.1.1. Differentially expressed genes (DEGs) between $CD37^{fl/fl}$ and $CD37^{-/-}$ AML LSCs were identified with *limma*. *ClusterProfiler* was applied to conduct functional enrichment analysis. The differential genes were further verified

by qPCR and Western blot.

Immunoblotting

Cells were lysed using RIPA lysis buffer (WEIAOBIO, WB0101) supplemented with 1:100 PMSF (Thermo, 36978B) and 1:100 phosphatase inhibitors (YAMEI, GRF102). The lysate was centrifuged and the supernatant was collected and subsequently boiled with 5xSDS protein loading buffer (YAMEI, LT101). Total proteins were separated by SDS-PAGE and transferred onto PVDF membranes (Millipore, IPVH00010), blocked with 5% skim milk dissolved in 1xTBS-T buffer (Solarbio, T1081), and incubated with the corresponding primary antibody at 4°C overnight. Subsequently, the membrane was washed in 1xTBS-T buffer and incubated with the corresponding secondary antibody conjugated with horseradish peroxidase (HRP) at room temperature for 1h. The membrane was then washed in 1xTBS-T buffer, incubated in hypersensitive ECL chemiluminescence reagent (Beyotime, P0018M) and imaged with ChemiScope 6000 (CLINX, 6100).

RNA extraction and quantitative real-time PCR

Total RNA was extracted from cells using Quick-RNA ™ Microprep Kit (ZYMO, R1050 & R1051). The RNA was reversely transcribed into cDNA with FastKing cDNA First-chain Synthesis Kit (TIANGEN, KR116).

Quantitative PCR (qPCR) was conducted on a Roche lightcycler96 Instrument in accordance with the manufacturer's instructions. Relative mRNA expression was quantified and normalized to the reference gene *GAPDH* for each sample. The qPCR primers were listed in Supplementary Table 1.

Immunofluorescence assay

Cells were fixed with 4% paraformaldehyde at 4°C for 2h and permeabilized with 0.2% Triton X-100 in PBS for 5min. Next, the cells were blocked with 5% BSA at room temperature for 1h and incubated with the corresponding primary antibody at 4°C overnight. After removal of unbound antibodies with PBS-T buffer, the cells were incubated with the corresponding secondary antibody conjugated with fluorochrome at room temperature for 1h. The cells were then washed with PBS-T buffer and incubated with DAPI for 5min. Finally, the cells were subjected to further observation and imaging under a fluorescence microscope.

Co-immunoprecipitation assay

Cells were lysed using Western and IP Cell Lysis Buffer (Beyotime, P0013) supplemented with 1:100 PMSF and 1:100 phosphatase inhibitors. The lysate was centrifuged and the supernatant was collected, boiled with 5xSDS protein loading buffer (as input) or incubated with anti-CD37

antibody (abcam, ab300400) or rabbit IgG (Beyotime, A7016) at 4℃ overnight (as IP or IgG). The IP and IgG samples were then incubated with protein A+G agarose (Beyotime, P2055) at 4℃ overnight. After removal of unbound antibodies with PBS buffer, the IP and IgG samples were boiled with 1xSDS protein loading buffer and subjected to further Western blot analysis.
