# Supplementary material for: CD37 regulates the self-renewal of leukemic stem cells via integrin-mediated signaling in acute myeloid leukemia
Source: Stem Cell Reports. 2025 Apr 17;20(5):102476. doi: 10.1016/j.stemcr.2025.102476 (PMC12143136; doi:10.1016/j.stemcr.2025.102476)
Supplement: Document S2. Article plus supplemental information [file mmc2.pdf]

Stem Cell Reports
Article
OPEN ACCESS

CD37 regulates the self-renewal of leukemic stem cells via integrin-mediated signaling in acute myeloid leukemia

Jinyuan Lu,[1,2,5] Lixin Lv,[1,2,5] Xiaoxue Tian,[1,2] Zheng Li,[3] Yuting Ma,[1,2] Nannan Li,[1,2] Jian Wang,[1,4] Guangming Wang,[4] Yu Zeng,[2] Wenjun Zhang,[2] Jun Xu,[4] and Aibin Liang[2,6,*]
[1]Tongji University School of Medicine, 500 Zhennan Road, Shanghai 200331, P.R. China
[2]Department of Hematology, Tongji Hospital, Tongji University School of Medicine, 389 Xincun Road, Shanghai 200333, P.R. China
[3]Department of Hematology, Renji Hospital, Shanghai Jiaotong University School of Medicine, 160 Pujian Road, Shanghai 201112, P.R. China
[4]Stem Cell Research Center, East Hospital, Tongji University School of Medicine, Shanghai 200331, P.R. China
[5]These authors contributed equally
[6]Lead contact
*Correspondence: lab7182@tongji.edu.cn
https://doi.org/10.1016/j.stemcr.2025.102476

SUMMARY

Leukemic stem cells (LSCs) are a small subset of leukemia cells that drive leukemia initiation and maintenance. Herein, we report that CD37, a member of transmembrane 4 superfamily (TM4SF), regulates the survival of acute myeloid leukemia (AML) cells as well as the self-renewal of AML LSCs. The downregulation of CD37 retarded proliferation and increased apoptosis in human AML cell lines THP-1 and OCI-AML2. Deficiency of CD37 *in vivo* had a minimal effect on normal hematopoiesis but significantly impeded leukemia maintenance and propagation, which led to increased apoptosis and decreased cell cycle entry in AML blasts as well as impaired colony formation and declined frequency of AML LSCs in the serial transplantation. Furthermore, CD37 interacted with integrin α4β7 and activated the phosphatidylinositol 3-kinase (PI3K)-AKT pathway mediated by integrin signaling. Our study provides novel insights for targeted therapy of AML, indicating CD37 as a safe and effective target for immunotherapy.

INTRODUCTION

Acute myeloid leukemia (AML) is a malignant clonal disorder characterized by excessive proliferation and expansion of myeloid blasts with abnormal differentiation, which leads to ineffective normal hematopoiesis, cytopenia, and transfusion dependency (DiNardo et al., 2023). As the most prevalent leukemia in elderly adults, AML accounts for over 80,000 deaths worldwide per year, and the number is still rising. In 1990, AML composed 18.0% of all cases of leukemia; by 2017, this proportion had increased to 23.1% (Dong et al., 2020). In China, the incidence of AML witnessed a significant increase by 1.54/100,000 from 1990 to 2017 (Dong et al., 2020). These data indicate that AML is still a nonnegligible concern that threatens human health.

Current therapeutic approaches for AML include a standard combination of cytarabine and anthracycline, which demonstrates a better efficacy for younger patients while exhibiting less effectiveness in elderly individuals. Recent emergence of novel reagents and targeted therapeutics shed a different light for the treatment of AML. For example, demethylating agents and venetoclax (BCL2 inhibitor) can be considered as alternative options for patients who are unable to tolerate intensive chemotherapy, while allo-hematopoietic stem cell transplantation (allo-HSCT) can be performed following the achievement of complete remission (Bhansali et al., 2023). However, persistent AML cells undetected by morphological analysis may survive inadequate post-remission treatment and form minimal residual disease (MRD), which leads to subsequent drug resistance and relapse (DiNardo et al., 2023).

Leukemic stem cells (LSCs) are functionally defined as leukemic cells that are capable of self-renewal and exhibit sustained survival under *ex vivo* conditions and engraftment into immunocompromised mice (Vetrie et al., 2020). Similar to hematopoietic stem cells (HSCs), LSCs self-renew to generate more LSCs and give rise to differentiated leukemia blasts. LSCs are responsible for the initiation, maintenance, and recurrence of AML (O'Reilly et al., 2021). In order to achieve durable remission in patients with AML, it is necessary to identify therapeutic targets for the elimination of LSCs. Membrane proteins upregulated on LSCs but not on HSCs or normal bone marrow (BM) cells, including CD25 (Saito et al., 2010), CD44 (Jin et al., 2006), and GPR56 (Pabst et al., 2016), were rendered as optimal targets to mark and eliminate LSCs residing in MRD. Meanwhile, other characteristics of LSCs, such as alterations in signal transduction (Wang et al., 2010), metabolic or epigenetic regulation (Nguyen et al., 2011; Sykes et al., 2016), and crosstalk with microenvironment (Mohle et al., 1998) can also be utilized for targeting LSCs.

Tetraspanins (TSPANs) are membrane proteins with 4 transmembrane helices. TSPANs do not mediate protein interactions through ligand-receptor binding; however, they can serve as scaffolding proteins and recruit a variety of partner proteins with specific functions, such as adhesion

© 2025 The Author(s).
Published by Elsevier Inc. on behalf of International Society for Stem Cell Research.
This is an open access article under the CC BY license (http://creativecommons.org/licenses/by/4.0/).

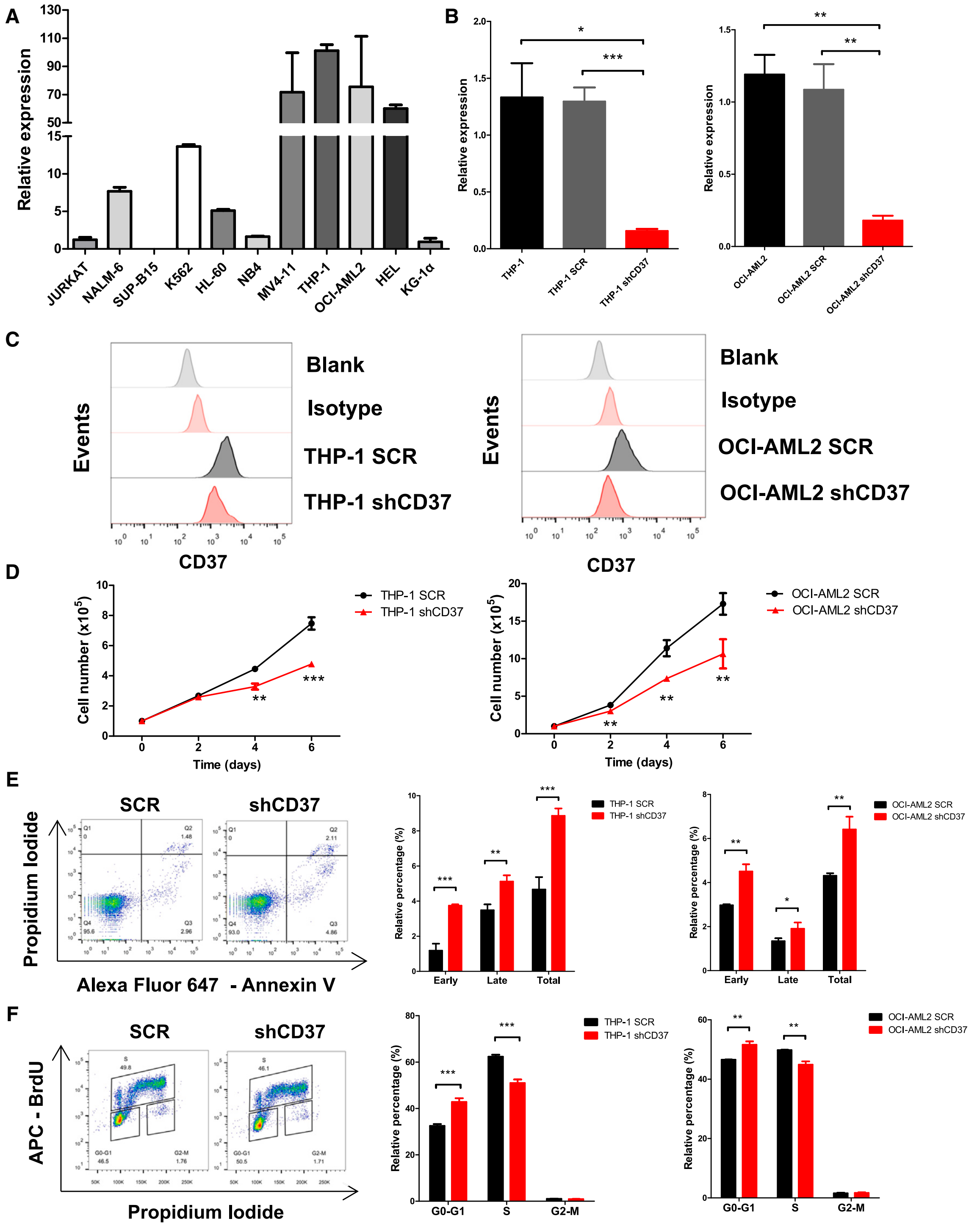

Figure 1. CD37 regulated the survival of human AML cell lines

(A) qPCR analysis for CD37 expression in various leukemia cell lines.

(B and C) qPCR and flow cytometric validation of CD37 knockdown efficiency in THP-1 and OCI-AML2 cells.

(D) Cell proliferation in THP-1 and OCI-AML2 cells transfected with SCR or shCD37.

(legend continued on next page)

molecules (e.g., integrins) and signaling molecules (e.g., tyrosine kinase receptors), to form a complex functional network known as the tetraspanin-enriched microdomain (TEM) (Detchokul et al., 2014) and mediate various biological processes including cell adhesion, migration, and signal transduction (Quagliano et al., 2023). Recent findings have unveiled a robust correlation between TSPANs and leukemia. The administration of antibodies targeting CD9 caused a significant reduction of $CD34^+$, $CD38^-$ LSCs (Kollmann et al., 2021). Knockdown of CD82 resulted in decreased phosphorylation of AKT and reduced expression of BCL2L12, which leads to increased apoptosis of AML cells and inhibited colony formation of AML LSCs (Nishioka et al., 2015).

CD37, a TSPAN predominantly expressed in the hematological system, exhibits high expression on mature B cells and is also present on T cells, monocytes, and natural killer (NK) cells (Pereira et al., 2015). In hematological malignancies, CD37 is upregulated in B cell non-Hodgkin's lymphoma (B-NHL), B cell acute lymphoblastic leukemia (B-ALL), and chronic lymphocytic leukemia (CLL) (Deckert et al., 2013; Scarfò et al., 2018). CD37 upregulation is also found in AML. A study revealed significantly higher CD37 expression on AML LSCs compared to normal HSCs (Pereira et al., 2015). Another study indicated that CD37 was absent on granulocyte-macrophage progenitors (GMPs) or common myeloid progenitors (CMPs), but reactivated following the transformation into LSCs, indicating a crucial role of CD37 in leukemic self-renewal (Wang et al., 2010). CD37 overexpression had been identified as a significant risk factor in AML, correlating with an unfavorable prognosis (Yan et al., 2021). CD37-positive AML cells were sensitive to IMGN529, an antibody-drug conjugate (ADC) targeting CD37, and the viability of AML cells declined in response to an increased concentration of IMGN529 (Larkin et al., 2018). Another ADC, AGS67E, had cytotoxic effects not only to B cell malignancies but also to AML cells, including $CD34^+$, $CD38^-$ AML LSCs (Pereira et al., 2015). In recent years, chimeric antigen receptor T (CAR-T) cells targeting CD37 have been developed, and CD37 CAR-T cells have demonstrated strong efficacy against CD37-expressing AML cells, with no observed cytotoxicity toward HSCs (Caulier et al., 2024). Taken together, the upregulation of CD37 on AML cells and AML LSCs underscores its potential as a promising therapeutic target for the treatment of AML.

While CD37 has attracted substantial attention in B cell-derived leukemia and lymphoma, there still remains a lack of foundational research to substantiate its role in AML. This study investigated the role of CD37 in AML initiation and maintenance as well as its impact on normal BM hematopoiesis, elucidating potential mechanisms by which CD37 regulated the survival of AML cells and the self-renewal of AML LSCs.

RESULTS

CD37 was upregulated in AML

We first conducted a comprehensive analysis of online databases to determine the expression profile of CD37 in various malignancies. CD37 is significantly upregulated in AML, compared to normal BM counterparts (Figures S1A and S1B). By categorizing patients with leukemia into distinct subtypes based on the French-American-British (FAB) classification (Bennett et al., 1976), we noticed a significant upregulation of CD37 in M4 and M5 AML (Figure S1C), and patients exhibiting high CD37 expression displayed an unfavorable prognosis (Figures S1C and S1D). Next, we assessed the expression of CD37 in a panel of leukemia cell lines available in our laboratory. The mRNA level of CD37 was highest in THP-1 (M5), OCI-AML2 (M4), MV4-11 (M5), and HEL (M6) but significantly lower in other AML cell lines (HL-60 and NB4), ALL cell lines (JURKAT, NAML-6, and SUP-B15), and chronic myeloid leukemia (CML) cell line (K562) (Figure 1A). We also investigated the expression of CD37 in AML patient samples and observed an upregulation of CD37 in $CD11b^+$ myeloid cells, compared to $CD11b^-$ cells (Figure S1E) and normal $CD11b^+$ BM cells (Figure S1F), indicating an upregulation of CD37 in the myeloid lineage. The aforementioned data suggest that CD37 was upregulated in AML and may serve as a therapeutic target.

CD37 knockdown inhibited the proliferation of human AML cell lines

To elucidate the function of CD37 in AML cells, we designed 2 short hairpin RNA (shRNA) plasmids specifically targeting CD37 (shCD37-1 and shCD37-2) and a scrambled plasmid (SCR) as a normal control. We chose THP-1 and OCI-AML2 as target cells since their expression of CD37 was highest among all cell lines. The cells were transfected with SCR or shCD37, and the knockdown efficacy of shCD37 was validated using qPCR and flow cytometry (Figures 1B, 1C, S2A, and S2B). CD37 deficiency retarded

(E) Representative flow cytometric analysis of apoptosis in THP-1 cells transfected with SCR or shCD37 (left). The proportions of early, late, and total apoptotic cells were also quantified (right).
(F) Representative flow cytometric analysis of cell cycle in THP-1 cells transfected with SCR or shCD37 (left). The proportions of THP-1 and OCI-AML2 cells in G0-G1 phase, S phase, or G2-M phase were also quantified (right). Error bars in (A), (B), (D), (E) and (F) were defined as mean ± SD. $*p < 0.05$, $**p < 0.01$, $***p < 0.001$.

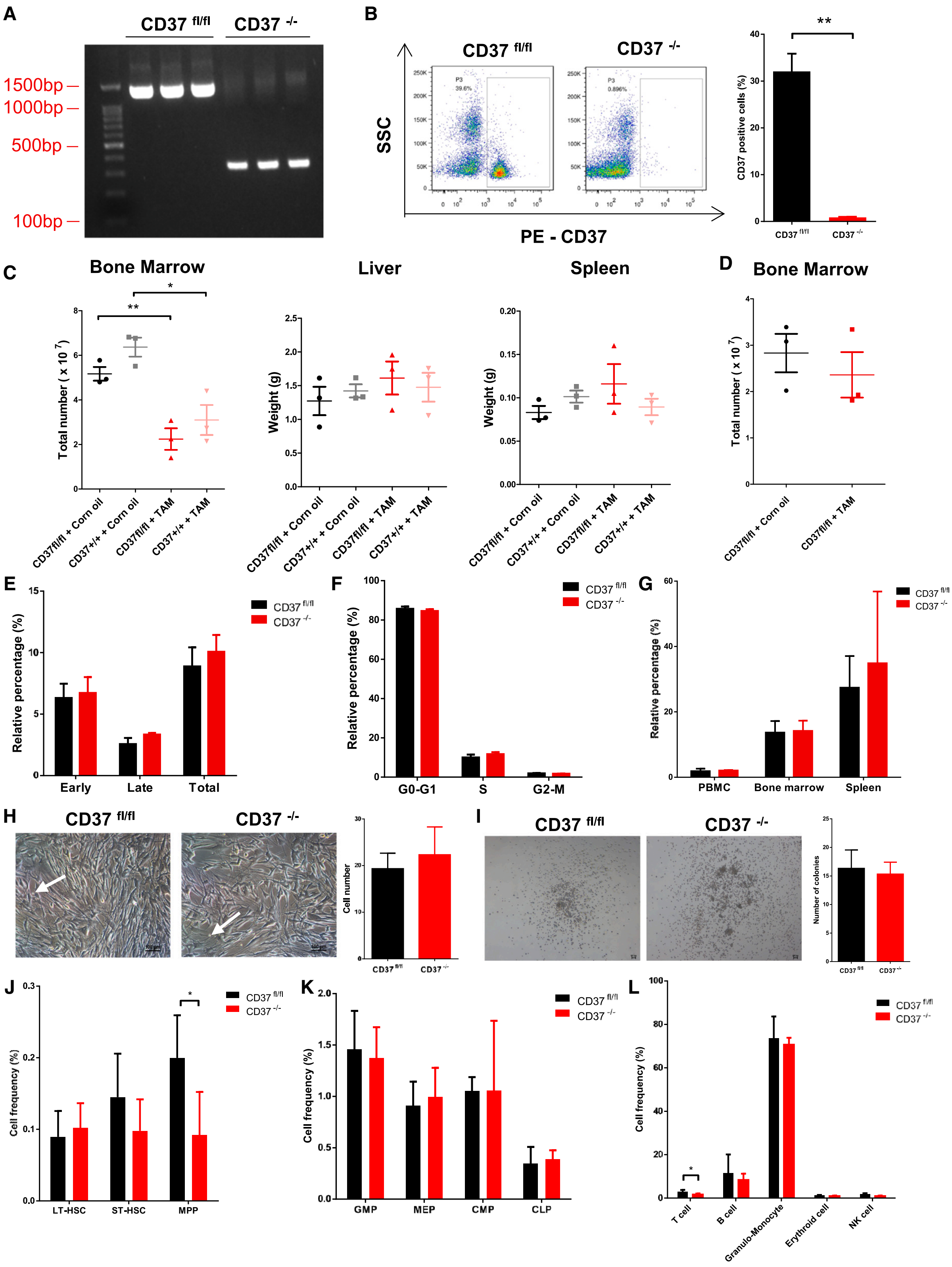

(legend on next page)

the proliferation of AML cells (Figures 1D and S2C). Meanwhile, more apoptotic cells were observed in CD37-deficient AML cells (Figures 1E and S2D). Knockdown of CD37 resulted in a G1-S cell-cycle arrest in THP-1 and OCI-AML2 cells, while the arrest was more significant using shCD37-1 (Figure 1F) compared to shCD37-2 (Figure S2E). The aforementioned data suggested a potential regulatory role of CD37 in the survival of AML cells.

CD37 deficiency had a minor effect on normal hematopoiesis

Next, we established a CD37 conditional knockout mouse model utilizing the CRISPR-Cas9 system. Generation and screening of $CD37^{fl/fl}$, Cre^{+} mice were performed as described in the methods section. Conditional knockout of CD37 (denoted as $CD37^{-/-}$) was induced through intraperitoneal injection of tamoxifen. The efficacy of CD37 deletion was confirmed by PCR and flow cytometry (Figures 2A and 2B).

We first investigated the direct impact of CD37 loss on normal BM cells. $CD37^{fl/fl}$, Cre^{+} mice and CD37 wild-type (WT) (denoted as $CD37^{+/+}$) mice were administrated with tamoxifen or corn oil. BM cells, livers, and spleens were harvested 1 month after injection. CD37 deletion resulted in a decrease in total BM cell number, while no significant difference was observed in liver weight and spleen weight (Figure 2C). To exclude potential inhibitory effects induced by tamoxifen, we extended the observation from 1 month to 4 months, and no significant difference in BM cell number was observed between $CD37^{fl/fl}$ and $CD37^{-/-}$ mice (Figure 2D). Meanwhile, CD37 deficiency did not affect apoptosis, cell cycle, homing, adhesion, or colony formation of normal BM cells (Figures 2E–2I), indicating that the impact of CD37 loss was transient and can be mitigated by compensatory mechanisms. Further validation using peripheral blood from $CD37^{fl/fl}$ and $CD37^{-/-}$ mice indicated that CD37 deficiency had a minimal impact on T cell, B cell, granulo-monocyte, and erythroid cell differentiation (Figure S3C). Apoptosis assay showed no statistical difference between NK cells from $CD37^{fl/fl}$ and $CD37^{-/-}$ mice at week 10 (Figure S3D). Another independent differentiation assay using BM cells from $CD37^{fl/fl}$ and $CD37^{-/-}$ mice showed that CD37 deficiency resulted in a decrease in the proportion of T cells and multipotent progenitors (MPPs), while exhibiting no impact on other cell types (Figures 2J–2L).

Based on these data, we further established a transplantation model and investigated the impact of CD37 loss on normal hematopoiesis over an extended duration. Lethally irradiated CD45.1 mice were transplanted with BM lineage-negative (Lin^{-}) cells derived from $CD37^{fl/fl}$, Cre^{+} mice (expressing CD45.2) and intraperitoneally injected with tamoxifen or corn oil 2 months post transplantation (Figure 3A). CD37 deletion did not affect the chimerism of CD45.1-expressing donor cells in the recipient mice (Figures 3B and 3C). A decreased proportion of T cells, B cells, and GMPs, as well as an increased proportion of granulo-monocytes, was observed in $CD37^{-/-}$ recipients at month 6 (Figures 3D–3F). In another transplantation assay, CD37 knockout was induced prior to transplantation, and the chimerism of donor cells remained comparable to the control group (Figures 3G–3I). CD37 deletion resulted in a decreased proportion of B cells, while no significant influence was observed on other cell types (Figures 3J–3L). In summary, CD37 deficiency exhibited only a marginal effect on normal hematopoiesis.

CD37 is dispensable for leukemogenesis but required for leukemia maintenance and self-renewal of LSCs

Given the limited impact of CD37 deletion on normal hematopoiesis, we thought to investigate the role of CD37 in AML initiation. In order to initiate AML, BM Lin^{-} cells obtained from $CD37^{fl/fl}$, Cre^{+} mice were transfected with MSCV-MLL-AF9-IRES-YFP (Figure S4A) *in vitro*. The YFP fluorescence was observed using an inverted microscope (Figure S4B), and

Figure 2. CD37 deficiency had a mild effect on normal BM cell function

(A) PCR validation of CD37 knockout efficiency.

(B) Flow cytometric validation of CD37 knockout efficiency.

(C) Total number of BM cells, liver weight, and spleen weight of $CD37^{fl/fl}$, Cre^{+} mice and CD37 WT mice 1 month after treatment with tamoxifen or corn oil ($n = 3$ for each).

(D) Total number of BM cells of $CD37^{fl/fl}$, Cre^{+} mice 4 months after treatment with tamoxifen or corn oil ($n = 3$).

(E and F) Quantification of apoptotic cells (E) and cell cycle distribution (F) in BM samples 1 month after treatment with tamoxifen or corn oil ($n = 3$ for each).

(G) Quantification of $CD45.2^{+}$ cells migrated to the peripheral blood, BM, or spleen of CD45.1 recipients 18 h after transplantation ($n = 4$).

(H) Representative images demonstrating the adherence of normal BM cells to OP9 stroma cells (left, scale bar, 100μm). The white arrows indicate adherent BM cells. The number of adherent BM cells was also quantified (right).

(I) Representative images demonstrating the colony formation of $CD37^{fl/fl}$ and $CD37^{-/-}$ BM Lin^{-} cells (left, scale bar, 50μm). The number of colonies was also quantified (right).

(J–L) Proportions of hematopoietic stem cells (J), progenitors (K), and mature cells (L) in total BM cells derived from $CD37^{fl/fl}$, Cre^{+} mice 1 month after treatment with tamoxifen or corn oil ($n = 4$ for each). Error bars in (B–L) were defined as mean ± SD. *$p < 0.05$, **$p < 0.01$.

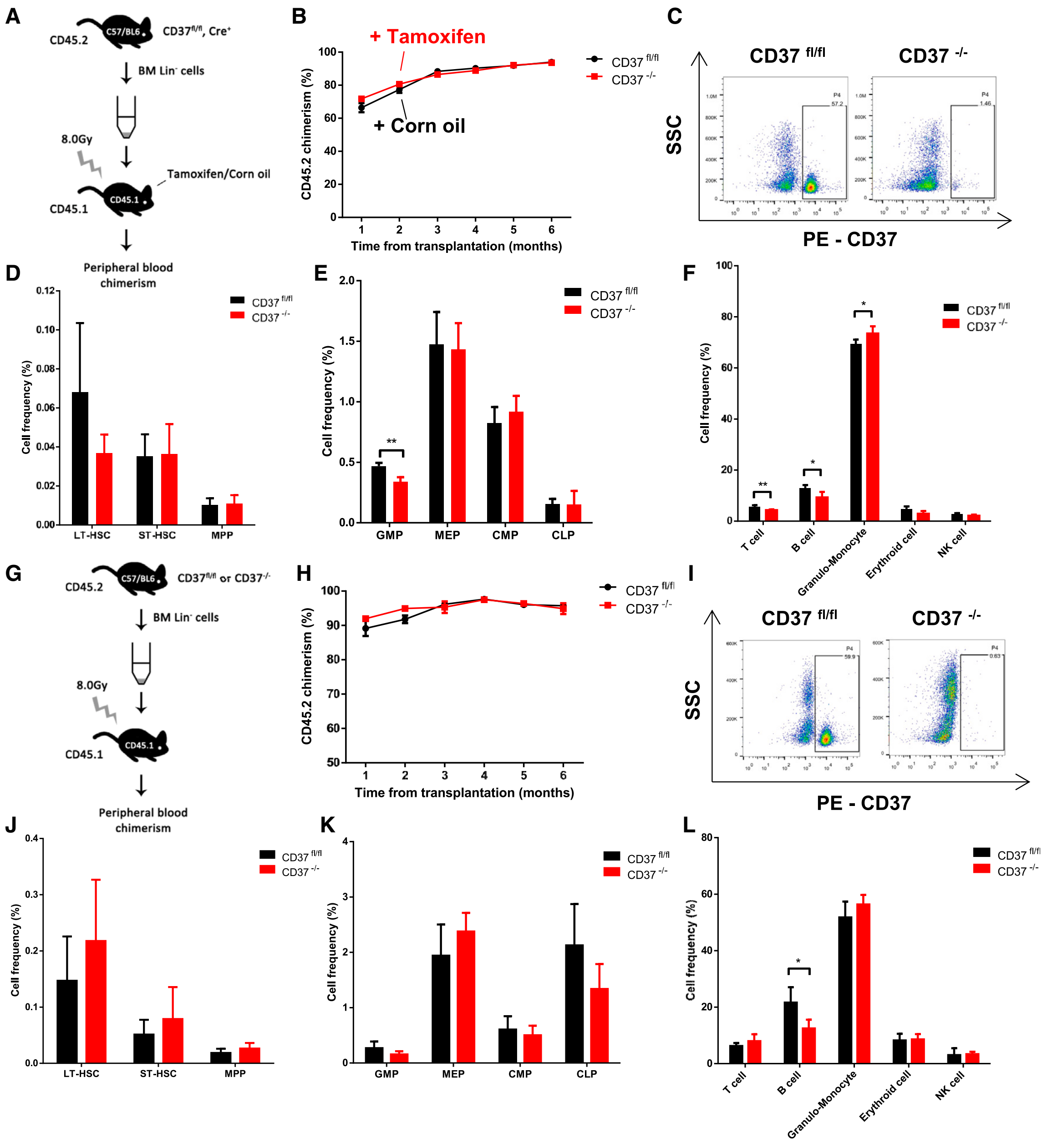

Figure 3. CD37 deficiency had a mild effect on normal hematopoiesis

(A and G) The schematic diagrams of normal transplantation model. (A) Tamoxifen or corn oil was administrated 2 months after transplantation. (G) Tamoxifen and corn oil were administrated prior to transplantation.

(B and H) Chimerism of CD45.2$^+$ cells in the peripheral blood of recipient mice (n = 4 for each).

(C and I) Representative flow cytometric analysis of CD37 expression in CD45.2$^+$ cells.

(D–F and J–L) Proportions of hematopoietic stem cells (D and J), progenitors (E and K), and mature cells (F and L) in CD45.2-expressing BM cells derived from CD37$^{fl/fl}$ or CD37$^{-/-}$ recipients (n = 4 for each). Error bars in (B), (D), (E), (F), (H), (J), (K) and (L) were defined as mean ± SD. *p < 0.05, **p < 0.01.

the transfection efficiency was assessed by PCR and flow cytometry (Figures S4C and S4D). The YFP$^+$ cells were subsequently sorted and transplanted into sublethally irradiated C57 mice (primary transplantation, Figure 4A). Conditional knockout of CD37 was administrated as described in the methods section. CD37 deletion did not accelerate or decelerate leukemogenesis of MLL-AF9 AML (Figure 4B). The extent of tumor infiltration was comparable between

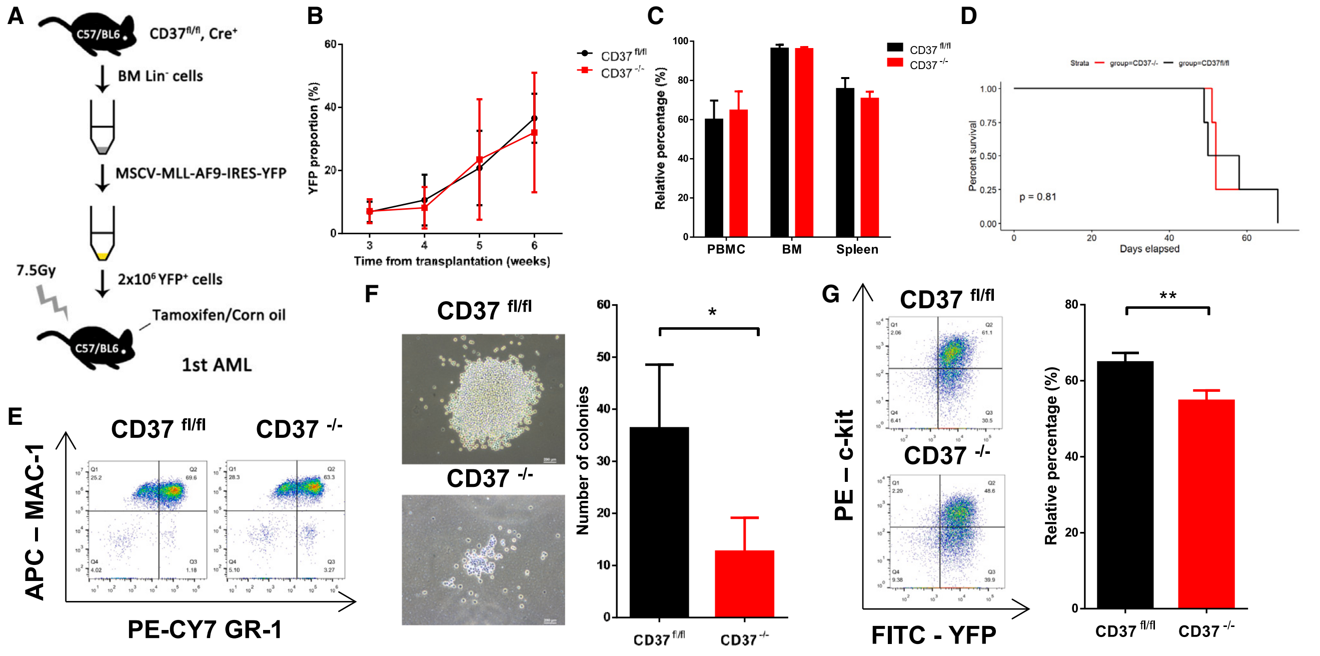

Figure 4. CD37 deficiency impaired the self-renewal of LSCs in the primary transplantation
(A) The schematic diagram of MLL-AF9 AML initiation.
(B) YFP^+ cell proportion in the peripheral blood of $CD37^{fl/fl}$ or $CD37^{-/-}$ recipients ($n = 4$).
(C) Infiltration of YFP^+ leukemia cells in the peripheral blood, bone marrow, and spleens ($n = 4$).
(D) Survival plot for $CD37^{fl/fl}$ and $CD37^{-/-}$ recipients ($n = 4$).
(E) Representative flow cytometric analysis of Mac-1 and Gr-1 expression in YFP^+ cells.
(F) Representative images demonstrating the colony formation of $CD37^{fl/fl}$ and $CD37^{-/-}$ AML LSCs (left, scale bar, 200μm). The number of colonies was also quantified (right).
(G) Representative flow cytometric analysis of YFP and c-kit expression in total BM cells (left). The proportion of c-kit^+ cells in YFP^+ cells was also quantified (right, $n = 4$). Error bars in (B), (C), (F) and (G) were defined as mean ± SD. $*p < 0.05$, $**p < 0.01$.

$CD37^{-/-}$ and $CD37^{fl/fl}$ mice, and the survival duration was similar in both groups (Figures 4C, 4D, and S4E–S4G). Over 90% of YFP^+ cells expressed Mac-1, and over 60% of YFP^+ cells co-expressed MAC-1 and GR-1 (Figure 4E), indicating development of myeloid leukemia. Although no statistical difference was observed in leukemogenesis, CD37 deletion significantly altered the functionality of AML LSCs. Following CD37 deletion, YFP^+, c-kit^+ AML cells (enriched for LSCs) exhibited a diminished capacity for colony formation (Figure 4F), and the proportion of c-kit^+ leukemia cells in the BM was reduced (Figure 4G), suggesting impaired self-renewal of $CD37^{-/-}$ AML LSCs.

To determine if continued AML maintenance requires CD37, we isolated YFP^+, c-kit^+ LSCs from premorbid AML mice and transplanted them into new recipients following irradiation (secondary transplantation, Figure 5A). Mice transplanted with $CD37^{-/-}$ LSCs exhibited a marked reduction in AML progression (Figure 5B), accompanied by reduced tumor infiltration (Figures 5C and S4H–S4J) and prolonged survival (Figure 5D), compared to those transplanted with $CD37^{fl/fl}$ LSCs. Moreover, $CD37^{-/-}$ leukemia cells demonstrated increased apoptosis (Figure 5E) and a G1-S arrest in the cell cycle (Figure 5F). We hypothesized if this phenotype was caused by variations in cell homing capability. No statistical differences were found in the ratio of $CD37^{-/-}$ or $CD37^{fl/fl}$ AML cells homed in peripheral blood, BM, or spleen (Figure 5G). Meanwhile, the adhesion of $CD37^{-/-}$ AML cells decreased slightly without reaching statistical difference (Figure 5H). The colony formation as well as the frequency of CD37-deficient AML LSCs was significantly reduced, compared to that of normal AML LSCs (Figures 5I and 5J).

Additionally, YFP^+, c-kit^+ LSCs derived from the secondary transplantation were further transplanted into new recipients after irradiation (tertiary transplantation, Figure 5A), and the tertiary AML exhibited a comparable but more pronounced phenotype. The infiltration of AML cells was significantly attenuated in mice transplanted with $CD37^{-/-}$ LSCs, leading to retarded progression of AML and extended survival of recipient mice (Figures 5K–5M and S4K–S4M). $CD37^{-/-}$ AML cells exhibited increased apoptosis (Figure 5N), a retarded cell cycle (Figure 5O),

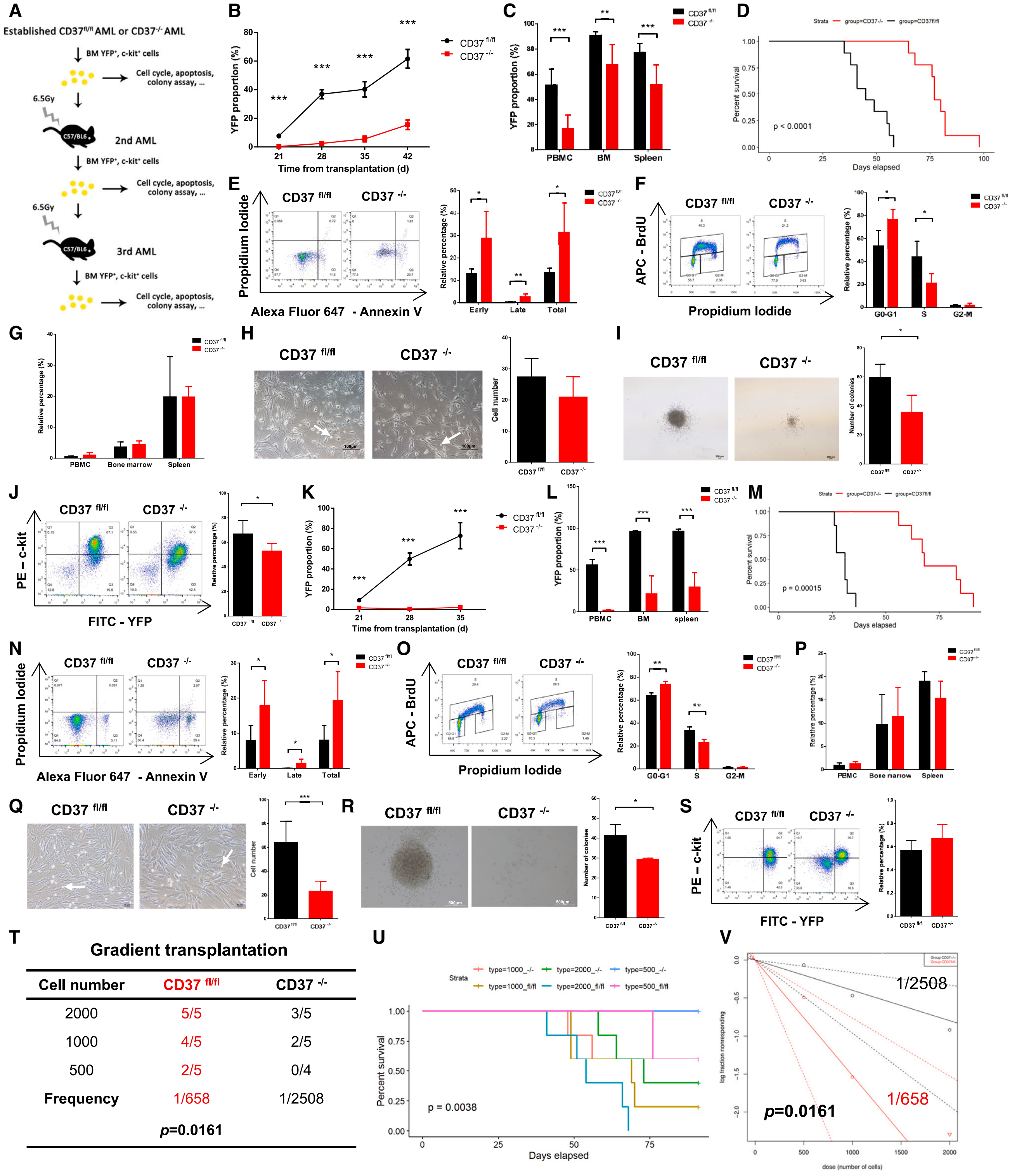

Figure 5. CD37 deficiency impaired leukemia maintenance and the self-renewal of LSCs in the secondary and tertiary transplantation

(A) The schematic diagram of continuous transplantation model for MLL-AF9 AML.

(B and K) YFP+ cell proportion in the peripheral blood of CD37$^{fl/fl}$ or CD37$^{-/-}$ recipients. (B) Secondary AML ($n = 9$); (K) tertiary AML ($n = 7$).

(legend continued on next page)

and decreased cell adhesion (Figure 5Q), without reaching significant difference in cell homing (Figure 5P). Moreover, LSCs lacking CD37 exhibited impaired colony formation (Figure 5R), while maintaining a comparable frequency to normal LSCs in the BM (Figure 5S). The aforementioned data indicated that CD37 is required for AML maintenance, and CD37 deficiency impaired the self-renewal of LSCs.

The expression of CD37 in AML LSCs was also investigated in the primary, secondary, and tertiary transplantation models. CD37 expression was significantly elevated in c-kit-high cells (Figures S5A–S5C). Simultaneously, c-kit was significantly enriched in the CD37-high population (Figures S5D–S5F), suggesting an association between CD37 expression and LSC function. This was further supported by the colony formation assay, where CD37 loss led to a reduction in both size and density of the colonies (Figures S5G–S5J).

To further elucidate the impact of CD37 loss on the self-renewal of LSCs, we established a gradient transplantation model and transplanted different numbers (2,000, 1,000, or 500) of $CD37^{fl/fl}$ or $CD37^{-/-}$ LSCs into recipient mice following irradiation. The frequency of LSCs was quantified using a limiting dilution assay (Hu and Smyth 2009). We found that CD37 deletion resulted in a significant decrease in the frequency of LSCs (1/2,508 vs. 1/658, Figures 5T–5V), demonstrating reduced self-renewal in $CD37^{-/-}$ LSCs.

Taken together, CD37 deficiency significantly impeded leukemia maintenance in MLL-AF9 AML, partially attributed to increased apoptosis, decreased cell cycle entry, and impaired self-renewal of AML LSCs.

CD37 deficiency led to reduced AKT phosphorylation and integrin expression

To gain a deeper understanding of the regulatory role of CD37 in MLL-AF9-induced AML, we sorted $CD37^{fl/fl}$ and $CD37^{-/-}$ AML LSCs and conducted transcriptome sequencing. 2,540 differentially expressed genes (DEGs) were identified and subjected to Kyoto Encyclopedia of Genes and Genomes (KEGG) functional enrichment analysis. Differential genes were enriched into 2 cellular functions: "transcriptional misregulation in cancer" and "DNA replication" (Figure 6A). Key DEGs associated with signal transduction, cell apoptosis, or cell cycle were subsequently selected and validated using qPCR. The comparison between RNA sequencing and qPCR data revealed a high level of consistency in the expression of *Ccnd2*, *Cdkn1b*, *Bcl2*, *Itgb7*, and *Pik3cb* among all identified DEGs (Figure 6B).

Subsequently, protein samples of $CD37^{fl/fl}$ and $CD37^{-/-}$ AML LSCs were utilized for western blot validation (The activation of phosphatidylinositol 3-kinase [PI3K]-AKT pathway was indicated by phosphorylated AKT, while the expression of *Cdkn1b* was determined by its translational product p27). AKT phosphorylation was significantly reduced in $CD37^{-/-}$ LSCs, along with the downregulation of BCL2 and ITGB7. Conversely, p27 expression was upregulated, while CCND2 expression remained unaltered in $CD37^{-/-}$ LSCs, compared to $CD37^{fl/fl}$ LSCs (Figure 6C).

We noticed that BCL2 and p27 were associated with the regulation of apoptosis and cell cycle, both of which were partially modulated by PI3K-AKT pathway (Darici et al., 2020). BCL2 is an anti-apoptotic protein whose downregulation leads to oligomerization of BAX and BAK and activation of caspase, therefore inducing cell apoptosis (Hata et al., 2015). P27, a cell cycle inhibitor, prevents cell cycle progression from the G1 phase to the S phase by interfering with the functions of cyclins and cyclin-dependent kinases (CDKs) (Bretones et al., 2015). In addition, integrin beta 7 (ITGB7), a member of the integrin family, acts as an adhesion molecule and regulates cell adhesion (Bachmann

(C and L) Infiltration of YFP^+ leukemia cells in the peripheral blood, bone marrow, and spleens. (C) Secondary AML (*n* = 8); (L) tertiary AML (*n* = 5).
(D and M) Survival plot for $CD37^{fl/fl}$ and $CD37^{-/-}$ recipients. (D) Secondary AML (*n* = 9); (M) tertiary AML (*n* = 7).
(E and N) Representative flow cytometric analysis of apoptosis in $CD37^{fl/fl}$ and $CD37^{-/-}$ AML cells (left) and quantification of early, late, and total apoptotic AML cells (right). (E) Secondary AML (*n* = 5); (N) tertiary AML (*n* = 5).
(F and O) Representative flow cytometric analysis of cell cycle in $CD37^{fl/fl}$ and $CD37^{-/-}$ AML cells (left) and quantification of AML cells in G0-G1 phase, S phase, or G2-M phase (right). (F) Secondary AML (*n* = 7); (O) tertiary AML (*n* = 4).
(G and P) Cell homing assay demonstrating the proportion of YFP^+ cells migrated to the peripheral blood, BM, or spleen of C57 recipients 18 h after transplantation. (G) Secondary AML (*n* = 5); (P) tertiary AML (*n* = 4).
(H and Q) Representative images demonstrating the adherence of AML cells to OP9 stroma cells (left, (H) scale bar, 100μm; (Q) scale bar, 50μm). The white arrows indicate adherent AML cells. The number of adherent AML cells was also quantified (right). (H) Secondary AML (*n* = 8); (Q) tertiary AML (*n* = 6).
(I and R) Representative images demonstrating the colony formation of $CD37^{fl/fl}$ and $CD37^{-/-}$ LSCs (left, (I) scale bar, 200μm; (R) scale bar, 500μm). The number of colonies was also quantified (right). (I) Secondary AML (*n* = 3); (R) tertiary AML (*n* = 3).
(J and S) Representative flow cytometric analysis of YFP and c-kit expression in total BM cells (left). The proportion of c-kit^+ cells in YFP^+ cells was also quantified (right). (J) Secondary AML (*n* = 6); (S) tertiary AML (*n* = 5).
(T–V) Recipient C57 mice were transplanted with 2,000, 1,000, or 500 AML LSCs. The survival data were recorded, and the frequency of AML LSCs was quantified using ELDA. Error bars in (B), (C), (E–L), (N–S) were defined as mean ± SD. $^*p < 0.05$, $^{**}p < 0.01$, $^{***}p < 0.001$.

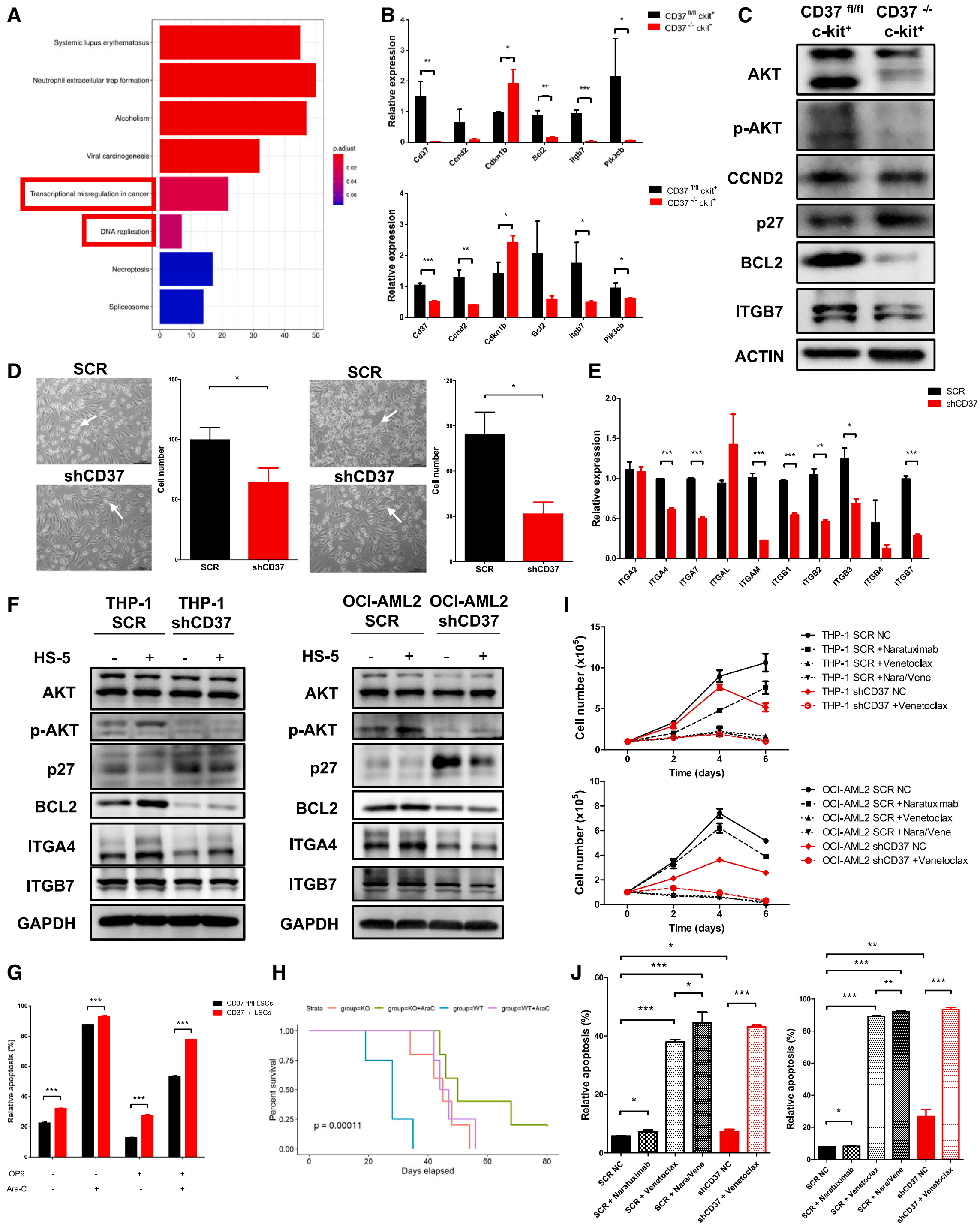

Figure 6. Transcriptomic analysis and verification of key DEGs

(A) KEGG analysis of DEGs identified between CD37$^{fl/fl}$ AML LSCs and CD37$^{-/-}$ AML LSCs.

(B and C) qPCR and western blot verification of key DEGs. (B) qPCR analysis. Upper, primary AML; lower, secondary AML; (C) western blot analysis, secondary AML.

(D) Representative images demonstrating the adherence of THP-1 cells (left) or OCI-AML2 cells (right, scale bar, 100μm) to HS-5 stroma cells. The white arrows indicate adherent AML cells. The number of adherent AML cells was also quantified.

(E) qPCR analysis for the expression of integrins on OCI-AML2 cells transfected with SCR or shCD37.

(legend continued on next page)

et al., 2019). Indeed, in the tertiary AML, the adhesion of AML cells was impaired following CD37 deletion (Figure 5Q). Similarly, THP-1 and OCI-AML2 cells with CD37 deficiency exhibited reduced adhesion when cocultured with the BM stroma cell HS-5, compared to their normal counterparts (Figure 6D). Integrins were heterodimeric transmembrane proteins consisting of an α subunit and a β subunit, and the initiation of their signaling occurred upon binding of integrins to their ligands, followed by the activation of multiple intracellular signaling pathways (Johansen et al., 2018; Floren et al., 2020). We wondered if CD37 regulated the survival of AML cells through integrin-mediated PI3K-AKT signaling. To validate this hypothesis, we first confirmed the impact of CD37 deficiency on integrin family expression. qPCR analysis revealed a significant downregulation of multiple integrins, including *ITGB7*, in CD37-deficient OCI-AML2 cells, either cultured alone or cocultured with HS-5 (Figures 6E and S6A). Moreover, the expression of integrin ligands on HS-5 was also reduced upon coculture with CD37-deficient OCI-AML2 cells (Figure S6B). Considering the differential expression of ITGB7 between $CD37^{fl/fl}$ and $CD37^{-/-}$ LSCs, we selected ITGB7 and its paired subunit ITGA4 as representatives to demonstrate the effect of CD37 deletion on integrin signaling. Western blot analysis of THP-1 and OCI-AML2 cells indicated that CD37 deficiency resulted in diminished phosphorylation of AKT, reduced expression of BCL2, ITGA4, and ITGB7, and elevated expression of p27 (Figure 6F). Moreover, when cocultured with HS-5, the level of AKT phosphorylation was significantly elevated in both THP-1 and OCI-AML2 cells, indicating the activation of the PI3K-AKT pathway within the stroma-mediated microenvironment (Figure 6F).

CD37 protects against chemotherapy and targeted immunotherapy

Next, the response of $CD37^{fl/fl}$ and $CD37^{-/-}$ AML cells to chemotherapy was also testified. When administrated with Ara-C, CD37-deficient AML LSCs co-cultured with stroma cells exhibited a significant increase in apoptosis, compared to normal AML LSCs (Figure 6G). In AML models, $CD37^{-/-}$ recipients treated with Ara-C exhibited the longest survival among all groups (Figure 6H). However, the survival benefit was not as long as anticipated, potentially attributable to the delayed initiation of Ara-C treatment (at day 7).

Finally, THP-1 and OCI-AML2 cells were treated with BCL2 inhibitor venetoclax and CD37 ADC naratuximab. Naratuximab alone induced a modest increase in apoptosis in THP-1 and OCI-AML2 cells, whereas the combination of venetoclax and naratuximab significantly enhanced apoptosis (Figures 6I and 6J). The application of venetoclax in CD37-deficient THP-1 and OCI-AML2 cells also demonstrated a synergistic pro-apoptotic effect (Figures 6I and 6J).

Collectively, these findings suggested that CD37 played a protective role within the BM microenvironment and might contribute to chemotherapy resistance. Targeting CD37 and its downstream effector BCL2 may enhance the anti-tumor efficacy, thereby improving the effectiveness of immunotherapy.

CD37 interacted with integrin α4β7

Previous studies have unveiled that TSPANs, including CD37, functioned as scaffolding proteins and facilitated the recruitment of signaling receptors and adhesion molecules to form TEM (Detchokul et al., 2014). We wondered if CD37 interacted with integrins on the cell membrane. Immunofluorescence staining indicated that CD37 was co-localized with integrin α4β7 on the membrane of THP-1 and OCI-AML2 cells (Figure S6C). In co-immunoprecipitation assay, the presence of ITGA4 and ITGB7 was verified in immunoprecipitation samples precipitated by anti-CD37 antibodies conjugated with protein A + G agarose (Figure S6D). A further verification using plasmids carrying exogenous CD37-FLAG, ITGA4-HIS, and ITGB7-HIS also indicated interactions between CD37-FLAG and ITGA4-HIS (ITGB7-HIS) (Figures S6E and S6F). In summary, CD37 interacted with integrin α4β7 on the cellular membrane of AML cells.

Overexpression of integrin α4β7 rescued the phenotypes caused by CD37 loss

Considering the co-localization and functional association between CD37 and integrin α4β7, we invested whether overexpression of ITGA4 (ITGA4 OE) or ITGB7 (ITGB7

(F) Immunoblotting assay for key DEGs in THP-1 and OCI-AML2 cells transfected with SCR or shCD37. The AML cells were cultured alone or with HS-5 stroma cells.
(G) Quantification of apoptotic YFP^+, c-kit^+ LSCs under different experimental conditions.
(H) Survival plot for recipient mice transplanted with $CD37^{fl/fl}$ or $CD37^{-/-}$ AML LSCs and received different treatments.
(I) Cell proliferation assay of THP-1 and OCI-AML2 cells transfected with SCR or shCD37 under different experimental conditions. Naratuximab, an ADC targeting CD37; venetoclax, a BCL2 inhibitor.
(J) Quantification of apoptotic cells under experimental conditions in (G) at day 6. Error bars in (B), (D), (E), (G), (I) and (J) were defined as mean ± SD. $^*p < 0.05$, $^{**}p < 0.01$, $^{***}p < 0.001$.

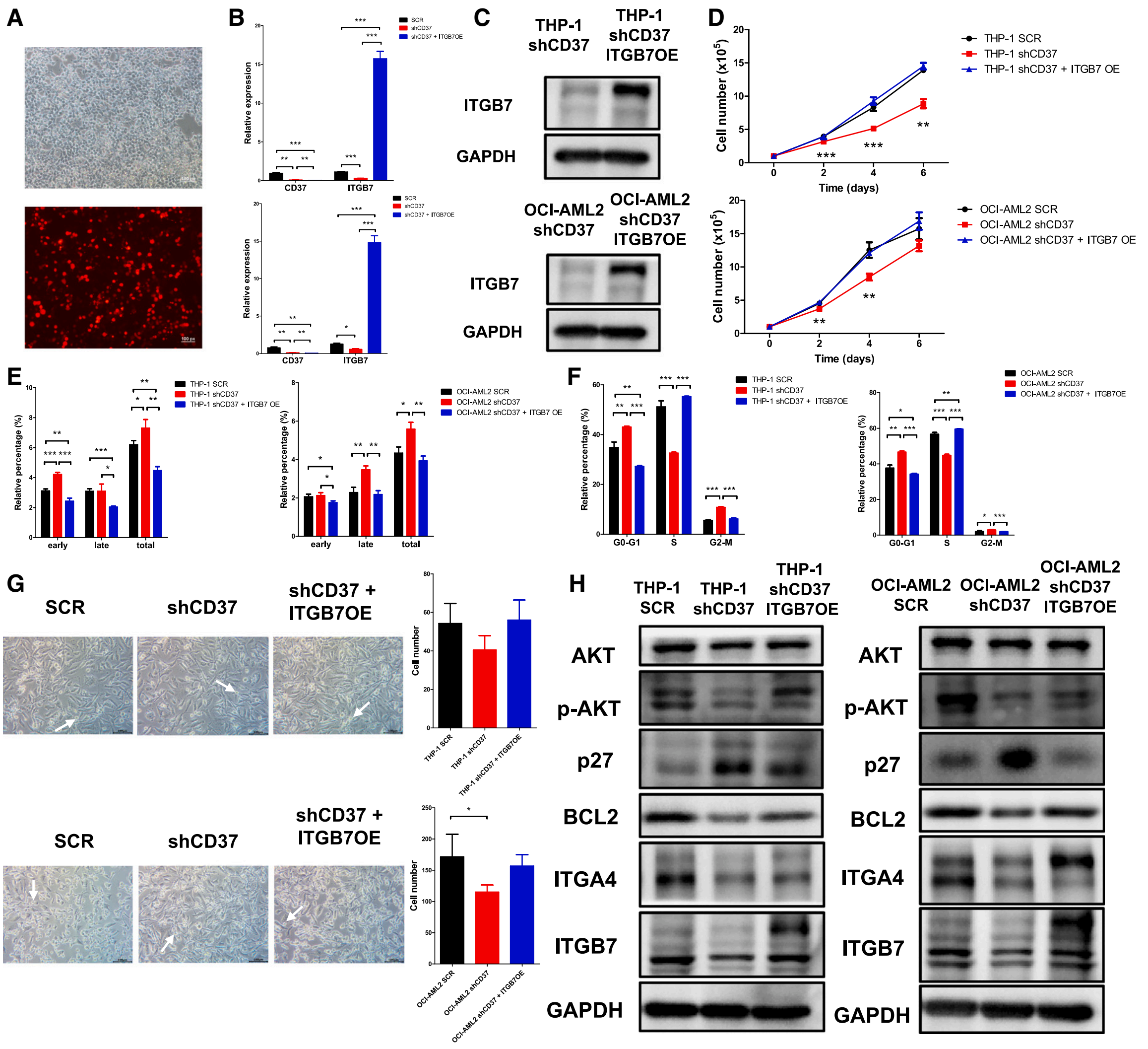

Figure 7. ITGB7 overexpression rescued the phenotypic effects caused by CD37 loss

(A–C) Fluorescence imaging, qPCR, and western blot validation of ITGB7 overexpression. (A) scale bar, 100μm.

(D) Cell proliferation in THP-1 and OCI-AML2 cells transfected with SCR, shCD37, or shCD37 + ITGB7 OE.

(E and F) Quantification for apoptosis (E) and cell cycle distribution (F) in THP-1 and OCI-AML2 cells transfected with SCR, shCD37, or shCD37 + ITGB7 OE.

(G) Representative images demonstrating the adherence of THP-1 cells (upper, scale bar, 100μm) or OCI-AML2 cells (lower, scale bar, 100μm) to HS-5 stroma cells. The white arrows indicate adherent AML cells. The number of adherent AML cells was also quantified.

(H) Immunoblotting for key DEGs in THP-1 and OCI-AML2 cells transfected with SCR, shCD37, or shCD37 + ITGB7 OE. Error bars in (B), (D), (E), (F) and (G) were defined as mean ± SD. *$p < 0.05$, **$p < 0.01$, ***$p < 0.001$.

OE) could rescue the phenotypic effects caused by CD37 loss. ITGA4 OE and ITGB7 OE) were validated through qPCR and western blot (Figures S7A–S7C and 7A–7C). ITGA4 OE, as well as ITGB7 OE, accelerated the proliferation of THP-1 and OCI-AML2 cells transfected with shCD37 (Figures S7D and 7D), reduced their apoptosis (Figures S7E and 7E), and promoted cell cycle progression to a similar extent as observed in cells transfected with SCR (Figures S7F and 7F). Additionally, cell adhesion was also enhanced in CD37-deficient AML cells overexpressing ITGA4 or ITGB7 (Figures S7G and 7G). Upregulation of ITGA4/ITGB7 compensated for the decrease in AKT phosphorylation and BCL2 expression, as well as the increase in p27 expression, caused by CD37 loss (Figures S7H and 7H). Collectively, integrin α4β7 played a crucial role in modulating the downstream targets of CD37, and overexpression of integrin α4β7 ameliorated the phenotypic consequences caused by CD37 loss.

DISCUSSION

A major challenge in treating AML is the persistence of LSCs, which possess self-renewal capacity and exhibit less sensitivity to conventional chemotherapy (Mitchell and Steidl 2020; O'Reilly et al., 2021). CD37, a membrane protein predominantly expressed in the hematological system, is upregulated in AML, and patients with elevated CD37 expression demonstrated an unfavorable prognosis (Yan et al., 2021). By conducting a comprehensive analysis of online databases and employing qPCR data in AML cell lines, we have observed a significant upregulation of CD37 in M4 and M5 subtypes of AML. We hypothesized that CD37 might exert a pivotal role in AML with the same morphological classification (e.g., MLL-AF9 AML, which belonged to M5 subtype). We selected THP-1 (M5) and OCI-AML2 (M4) for further investigation, as their expression of CD37 was highest among all cell lines. Our *in vitro* studies provided compelling evidences demonstrating the pro-apoptotic effects of shRNA targeting CD37 in THP-1 and OCI-AML2 cells. These findings were consistent with previous studies identifying CD37 as a risk factor in AML (Yan et al., 2021). The diminished viability observed in AML cells transfected with shCD37 underscored CD37 as a promising therapeutic approach for the treatment of AML.

For *in vivo* studies, the absence of CD37 resulted in only marginal effects on normal hematopoiesis. The primary impact of CD37 loss was the alterations in mature cell differentiation. However, these alterations were mild and had no significant impact on hematopoietic reconstitution. In contrast, CD37 deletion significantly diminished the capacity of AML LSCs to repopulate and maintain leukemia upon continuous transplantation. $CD37^{-/-}$ AML cells exhibited increased apoptosis, retarded cell cycle, and reduced adhesion to stroma cells, compared to $CD37^{fl/fl}$ AML cells. CD37 deficiency resulted in decreased colony formation of AML LSCs on methylcellulose and reduced frequency of LSCs within leukemia blasts. These findings highlighted the regulatory role of CD37 in modulating the self-renewal of AML LSCs. The absence of statistical differences in the initial AML induction might attribute to imbalanced transfection efficacy of MSCV-MLL-AF9-IRES-YFP into Lin^{-} cells, which comprised hematopoietic cells at different stages and possess uneven self-renewal capabilities. One approach is to transplant a fixed number of YFP^{+} c-kit^{+} LSCs into new recipients, thereby standardizing the initial leukemic burden to ensure comparability in subsequent secondary and tertiary leukemia models.

Transcriptome sequencing and subsequent qPCR and western blot validation indicated that CD37 deficiency in AML LSCs leads to reduced AKT phosphorylation, decreased BCL2 expression, and increased p27 expression, which was consistent with the pro-apoptotic phenotype observed in $CD37^{-/-}$ AML LSCs. Furthermore, CD37 knockdown in AML cell lines resulted in decreased activation of PI3K-AKT pathway and reduced expression of various integrins, including ITGA4 and ITGB7. Immunofluorescence assay and coimmunoprecipitation experiments confirmed the existence of protein-protein interactions between CD37 and integrin α4β7. Overexpression of integrin α4β7 in AML cells transfected with shCD37 compensated for the anti-proliferative effect caused by CD37 loss, suggesting that CD37 regulates its downstream targets partially through integrin-mediated PI3K-AKT signaling.

The interactions between TSPANs and partner proteins in TEM had been extensively documented in various studies. For example, CD82 could regulate the localization of β1 integrins on the cell membrane and activate subcellular p38-MAPK pathway, thereby promoting AML cell survival (Floren et al., 2020). The loss of CD37 in neutrophils resulted in enhanced internalization of leukocyte function-associated antigen (LFA)-1 and reduced actin accumulation, thereby disturbing neutrophil adhesion and migration (Wee et al., 2015). Our findings demonstrated that CD37 not only interacted with integrins but also regulated integrin expression at the transcriptional level. Previous studies have also indicated that CD37 itself could participate in the regulation of downstream targets, such as SHP-1, SYK, and GSK3β, through its cytoplasmic tails (Lapalombella et al., 2012), suggesting diverse regulatory mechanisms. Further investigations are needed to identify pathways and transcriptional regulators implicated in CD37-mediated processes.

Persistent activation of PI3K-AKT pathway was observed in 50%–80% of individuals with AML, accompanied by a reduction in overall survival (Park et al., 2009). The substrates of AKT played a crucial role in regulating cell cycle, proliferation, and metabolism (Bertacchini et al., 2015; Manning and Toker 2017). For instance, patients with AML harboring FLT3-LTD mutation exhibited pronounced activation of AKT and subsequent phosphorylation of FOXO3a, which inhibited the expression of p27 and the pro-apoptotic protein Bcl-2 interacting mediator of cell death (BIM), facilitating cell cycle progression (Brandts et al., 2005). PI3K-AKT pathway also played a pivotal role in the regulation of MLL-AF9-induced AML and the survival of AML LSCs. For example, deficiency of S6K1, a downstream effector of mTORC1, enhanced the survival of recipient mice transplanted with MLL-AF9 LSCs (Ghosh et al., 2016). Inhibition of HDAC3, a facilitator of AKT phosphorylation, significantly impeded AML progression

and enhanced the susceptibility of MLL-AF9-positive AML cells to chemotherapy (Long et al., 2017). Collectively, these studies provided compelling evidences for the pivotal role of PI3K-AKT pathway in the regulation of AML cell survival.

Taken together, our findings demonstrate a regulatory role of CD37 in MLL-AF9-induced AML, in which CD37 interacts with integrin family members (e.g., α4β7) and governs the survival of AML cells as well as the self-renewal of AML LSCs through integrin-mediated PI3K-AKT-BCL2/P27 signaling. The regulatory mechanism of CD37 is summarized in Figure S7I. Our study shed a different light for targeted therapy of AML, indicating CD37 as a safe and promising target for immunotherapy. However, there were still some limitations. For instance, the clinical sample size was limited, and the mechanisms underlying CD37-integrin interactions were not fully elucidated. Moreover, the *in vivo* chemotherapy experiments require additional replication. Further studies are essential to comprehensively assess the efficacy and safety of CD37-targeted immunotherapy in AML.

METHODS

Method details

Cell culture

THP-1, OCI-AML2, MV4-11, K562, NALM-6, JURKAT, KG-1α, and NB4 were cultured in RPMI 1640 medium (Gibco, 11875-093) with 10% fetal bovine serum (FBS) (BI, 04-001-1A) and 1% penicillin/streptomycin (Gibco, 15140-122). HEL, SUP-B15, and HL-60 were cultured in IMDM medium (Gibco, 12440-061) with 10% FBS and 1% penicillin-streptomycin (PS). OP9 was maintained in MEM-α (Gibco, 12561-056) with 20% FBS and 1% PS. 293T and HS-5 were maintained in DMEM (Gibco, 11965-092) containing 10% FBS and 1% PS. All cell lines were tested as mycoplasma negative.

Lentiviral infection

shRNA sequences were cloned into pLVX-shRNA vector (Clontech Laboratories). 293T cells were transfected with shRNA plasmids together with Delta-89 and VSVG (Addgene) using Lipo293 Transfection Reagent (Beyotime, C0521). The supernatant was collected 48 h after transfection, strained with 0.45 μm filter (Millipore, SLHVR33RB), mixed with polybrene (10 μg/mL, 1:1000), and added to target cells for centrifugation of 2 h. The cells were cultured at 37°C overnight and re-infected with the supernatant collected 72 h after transfection. The target cells were purified with puromycin (1 mg/mL, 1:1,000) and subjected to further analysis.

Generation of experimental mice

For conditional knockout of CD37, 8-week-old $CD37^{fl/fl}$, Cre^+ mice were intraperitoneally injected with tamoxifen (Solarbio, 10540-29-1) resolved in corn oil (20 mg/kg) for 5 consecutive days. For genotyping analysis, Quick Genotyping Assay Kit (Beyotime, D7283M) was applied to extract genomic DNA from mice tail. The genotypes of the experimental mice were verified by PCR, and the primers are listed in Table S2.

Transplantation assay for normal hematopoiesis

For BM transplantation, lineage-negative (Lin^-) BM cells were obtained from $CD37^{fl/fl}$, Cre^+ mice or $CD37^{-/-}$ mice and purified with magnetic beads (Miltenyi, 130-090-858), followed by transplantation into lethally irradiated (8.0 Gy) CD45.1 mice. The peripheral blood of recipient mice was collected monthly post transplantation, and the frequency of CD45.2-positive cells was quantified by flow cytometry. The recipients were euthanized 6 months after transplantation, and the BM cells were labeled with flow cytometry antibodies for differentiation assays. For conditional knockout of CD37, the experimental methods were mentioned earlier.

Generation of leukemia models

To initiate MLL-AF9 leukemia, Lin^- BM cells were obtained from $CD37^{fl/fl}$, Cre^+ mice and retrovirally infected with MSCV-MLL-AF9-IRES-YFP and PCL-ECO (Addgene). The Lin^- cells were cultured in RPMI 1640 medium with 20% FBS, 1% PS, 10 ng/mL mouse interleukin (IL)-3, IL-6, stem cell factor (SCF), and granulocyte colony stimulating factor (G-CSF). YFP^+ Lin^- cells were harvested 48 h after infection and transplanted into sublethally irradiated (7.5 Gy) C57 mice through intravenous injection. Tamoxifen or corn oil was intraperitoneally administrated into recipient mice 7 days after transplantation. Peripheral blood of recipient mice was collected weekly following transplantation, and the proportion of YFP^+ cells was analyzed by flow cytometry. Premorbid mice were sacrificed, and relevant tissues were harvested for subsequent analysis. For continuous transplantation, YFP^+, c-kit^+ cells from established leukemia were sorted and transplanted into sublethally irradiated (6.5 Gy) new recipient C57 mice (4,000 YFP^+, c-kit^+ cells per mouse) together with sufficient BM-supporting cells. For the gradient transplantation model, 2,000, 1,000, or 500 YFP^+, c-kit^+ cells together with sufficient BM-supporting cells were transplanted into sublethally irradiated (6.5 Gy) recipient C57 mice, and the frequency of LSCs was determined using the online software ELDA (Hu and Smyth 2009). For homing assays, 5×10^6 YFP^+ cells were transplanted into sublethally irradiated (7.5 Gy) C57 mice. The recipients were euthanized 18–20 h post transplantation for subsequent analysis. For Ara-C treatment, 200 mg/kg Ara-C was intravenously injected into recipients 1 week after transplantation of $CD37^{fl/fl}$ or $CD37^{-/-}$ AML LSCs, and the treatment was continued for 5 consecutive days. 0.9% saline was taken as negative control.

Colony formation assay

1,000 YFP$^+$, c-kit$^+$ cells isolated from leukemia mice transplanted with CD37$^{fl/fl}$ or CD37$^{-/-}$ AML LSCs were plated on methylcellulose medium (STEMCELL, M3434) in a 6-well plate and incubated at 37°C for 10–14 days. The number of colonies was quantified using an inverted microscope. The size and density of colonies were analyzed using FlowJo.

Statistics

All data were obtained from independent experiments. Quantitative data were represented as mean ± SD. Statistical differences between two independent groups were assessed using unpaired 2-tailed Student's t test. Mann-Whitney U test was used to compare differences between nonparametric data. One-way analysis of variance was applied to assess differences among multiple groups. Log rank test was employed to compare differences in Kaplan-Meier survival analysis, and the survival curves were depicted in R 4.1.1. Flow cytometry data were analyzed in FlowJo 10.4. The histograms and line charts were generated using GraphPad Prism 8. "*n*" in the figure legends indicated the number of mice. $p < 0.05$ was considered as statistically significant (*$p < 0.05$; **$p < 0.01$; ***$p < 0.001$).

RESOURCE AVAILABILITY

Lead contact

Further information and requests for resources and reagents should be directed to and will be fulfilled by the lead contact, Aibin Liang (lab7182@tongji.edu.cn).

Materials availability

All unique/stable reagents generated in the current study are available from the lead contact on reasonable request.

Data and code availability

The data and codes generated in the current study are available from the lead contact on reasonable request. The accession number for the transcriptome sequencing reported in this paper is GEO: GSE292186.

ACKNOWLEDGMENTS

The author would like to thank Professor Yi Sun (Tongji Hospital, Shanghai, China) for providing kind suggestions on article layout and J.X. for providing experimental facilities. This study was partially supported by the National Natural Science Foundation of China (grant no. 81770151).

AUTHOR CONTRIBUTIONS

J.L. designed the research, performed the experiments, analyzed the data, and drafted the manuscript. L.L. designed the research, performed part of the experiments, and analyzed the data. W.Z., G.W., J.X., and A.L. provided materials, designed the research, and revised the manuscript. Z.L., X.T., Y.M., and N.L. provided materials and performed part of the experiments. J.W. and Y.Z. provided materials and suggestions for the experiments. All authors have read and approved the final manuscript.

DECLARATION OF INTERESTS

The authors declare no competing interests.

SUPPLEMENTAL INFORMATION

Supplemental information can be found online at https://doi.org/10.1016/j.stemcr.2025.102476.

Received: August 14, 2024
Revised: March 18, 2025
Accepted: March 18, 2025
Published: April 17, 2025

REFERENCES

Bachmann, M., Kukkurainen, S., Hytönen, V.P., and Wehrle-Haller, B. (2019). Cell Adhesion by Integrins. Physiol. Rev. *99*, 1655–1699.

Bennett, J.M., Catovsky, D., Daniel, M.-T., Flandrin, G., Galton, D.A., Gralnick, H.R., and Sultan, C. (1976). Proposals for the Classification of the Acute Leukaemias French-American-British (FAB) Co-operative Group. Br. J. Haematol. *33*, 451–458.

Bertacchini, J., Heidari, N., Mediani, L., Capitani, S., Shahjahani, M., Ahmadzadeh, A., and Saki, N. (2015). Targeting PI3K/AKT/mTOR network for treatment of leukemia. Cell. Mol. Life Sci. *72*, 2337–2347.

Bhansali, R.S., Pratz, K.W., and Lai, C. (2023). Recent advances in targeted therapies in acute myeloid leukemia. J. Hematol. Oncol. *16*, 29.

Brandts, C.H., Sargin, B., Rode, M., Biermann, C., Lindtner, B., Schwäble, J., Buerger, H., Müller-Tidow, C., Choudhary, C., McMahon, M., et al. (2005). Constitutive Activation of Akt by Flt3 Internal Tandem Duplications Is Necessary for Increased Survival, Proliferation, and Myeloid Transformation. Cancer Res. *65*, 9643–9650.

Bretones, G., Delgado, M.D., and León, J. (2015). Myc and cell cycle control. Biochimica et Biophysica Acta (BBA) - Gene Regulatory Mechanisms *1849*, 506–516.

Caulier, B., Joaquina, S., Gelebart, P., Dowling, T.H., Kaveh, F., Thomas, M., Tandaric, L., Wernhoff, P., Katyayini, N.U., Wogsland, C., et al. (2024). CD37 is a safe chimeric antigen receptor target to treat acute myeloid leukemia. Cell Rep. Med. *5*, 101572.

Darici, S., Alkhaldi, H., Horne, G., Jørgensen, H.G., Marmiroli, S., and Huang, X. (2020). Targeting PI3K/Akt/mTOR in AML: Rationale and Clinical Evidence. J. Clin. Med. *9*, 2934.

Deckert, J., Park, P.U., Chicklas, S., Yi, Y., Li, M., Lai, K.C., Mayo, M.F., Carrigan, C.N., Erickson, H.K., Pinkas, J., et al. (2013). A novel anti-CD37 antibody-drug conjugate with multiple anti-tumor mechanisms for the treatment of B-cell malignancies. Blood *122*, 3500–3510.

Detchokul, S., Williams, E.D., Parker, M.W., and Frauman, A.G. (2014). Tetraspanins as regulators of the tumour microenvironment: implications for metastasis and therapeutic strategies. Br. J. Pharmacol. *171*, 5462–5490.

DiNardo, C.D., Erba, H.P., Freeman, S.D., and Wei, A.H. (2023). Acute myeloid leukaemia. Lancet *401*, 2073–2086.

Dong, Y., Shi, O., Zeng, Q., Lu, X., Wang, W., Li, Y., and Wang, Q. (2020). Leukemia incidence trends at the global, regional, and national level between 1990 and 2017. Exp. Hematol. Oncol. *9*, 14.

Floren, M., Restrepo Cruz, S., Termini, C.M., Marjon, K.D., Lidke, K.A., and Gillette, J.M. (2020). Tetraspanin CD82 drives acute myeloid leukemia chemoresistance by modulating protein kinase C alpha and β1 integrin activation. Oncogene *39*, 3910–3925.

Ghosh, J., Kobayashi, M., Ramdas, B., Chatterjee, A., Ma, P., Mali, R.S., Carlesso, N., Liu, Y., Plas, D.R., Chan, R.J., and Kapur, R. (2016). S6K1 regulates hematopoietic stem cell self-renewal and leukemia maintenance. J. Clin. Investig. *126*, 2621–2625.

Hata, A.N., Engelman, J.A., and Faber, A.C. (2015). The BCL2 Family: Key Mediators of the Apoptotic Response to Targeted Anticancer Therapeutics. Cancer Discov. *5*, 475–487.

Hu, Y., and Smyth, G.K. (2009). ELDA: Extreme limiting dilution analysis for comparing depleted and enriched populations in stem cell and other assays. J. Immunol. Methods *347*, 70–78.

Jin, L., Hope, K.J., Zhai, Q., Smadja-Joffe, F., and Dick, J.E. (2006). Targeting of CD44 eradicates human acute myeloid leukemic stem cells. Nat. Med. *12*, 1167–1174.

Johansen, S., Brenner, A.K., Bartaula-Brevik, S., Reikvam, H., and Bruserud, Ø. (2018). The Possible Importance of 3 Integrins for Leukemogenesis and Chemoresistance in Acute Myeloid Leukemia. Int. J. Mol. Sci. *19*, 251.

Kollmann, S., Grausenburger, R., Klampfl, T., Prchal-Murphy, M., Bastl, K., Pisa, H., Knab, V.M., Brandstoetter, T., Doma, E., Sperr, W.R., et al. (2021). A STAT5B-CD9 axis determines self-renewal in hematopoietic and leukemic stem cells. Blood *138*, 2347–2359.

Lapalombella, R., Yeh, Y.-Y., Wang, L., Ramanunni, A., Rafiq, S., Jha, S., Staubli, J., Lucas, D.M., Mani, R., Herman, S.E.M., et al. (2012). Tetraspanin CD37 Directly Mediates Transduction of Survival and Apoptotic Signals. Cancer Cell *21*, 694–708.

Larkin, K., Guth, E., Harrington, B.K., Grieselhuber, N., Muthusamy, N., and Byrd, J.C. (2018). CD37 Expression in Acute Myeloid Leukemia Provides New Target for Directed Therapy. Blood *132*, 4056.

Long, J., Fang, W.Y., Chang, L., Gao, W.H., Shen, Y., Jia, M.Y., Zhang, Y.X., Wang, Y., Dou, H.B., Zhang, W.J., et al. (2017). Targeting HDAC3, a new partner protein of AKT in the reversal of chemoresistance in acute myeloid leukemia via DNA damage response. Leukemia *31*, 2761–2770.

Manning, B.D., and Toker, A. (2017). AKT/PKB Signaling: Navigating the Network. Cell *169*, 381–405.

Mitchell, K., and Steidl, U. (2020). Targeting Immunophenotypic Markers on Leukemic Stem Cells: How Lessons from Current Approaches and Advances in the Leukemia Stem Cell (LSC) Model Can Inform Better Strategies for Treating Acute Myeloid Leukemia (AML). Cold Spring Harb. Perspect. Med. *10*, a036251.

Mohle, R., Bautz, F., Rafii, S., Moore, M.A.S., Brugger, W., and Kanz, L. (1998). The chemokine receptor CXCR-4 is expressed on CD34+ hematopoietic progenitors and leukemic cells and mediates transendothelial migration induced by stromal cell-derived factor-1. Blood *91*, 4523–4530.

Nguyen, A.T., Taranova, O., He, J., and Zhang, Y. (2011). DOT1L, the H3K79 methyltransferase, is required for MLL-AF9–mediated leukemogenesis. Blood *117*, 6912–6922.

Nishioka, C., Ikezoe, T., Takeuchi, A., Nobumoto, A., Tsuda, M., and Yokoyama, A. (2015). The novel function of CD82 and its impact on BCL2L12 via AKT/STAT5 signal pathway in acute myelogenous leukemia cells. Leukemia *29*, 2296–2306.

O'Reilly, E., Zeinabad, H.A., and Szegezdi, E. (2021). Hematopoietic versus leukemic stem cell quiescence: Challenges and therapeutic opportunities. Blood Rev. *50*, 100850.

Pabst, C., Bergeron, A., Lavallée, V.-P., Yeh, J., Gendron, P., Norddahl, G.L., Krosl, J., Boivin, I., Deneault, E., Simard, J., et al. (2016). GPR56 identifies primary human acute myeloid leukemia cells with high repopulating potential in vivo. Blood *127*, 2018–2027.

Park, S., Chapuis, N., Tamburini, J., Bardet, V., Cornillet-Lefebvre, P., Willems, L., Green, A., Mayeux, P., Lacombe, C., and Bouscary, D. (2010). Role of the PI3K/AKT and mTOR signaling pathways in acute myeloid leukemia. Haematologica *95*, 819–828.

Pereira, D.S., Guevara, C.I., Jin, L., Mbong, N., Verlinsky, A., Hsu, S.J., Aviña, H., Karki, S., Abad, J.D., Yang, P., et al. (2015). AGS67E, an Anti-CD37 Monomethyl Auristatin E Antibody–Drug Conjugate as a Potential Therapeutic for B/T-Cell Malignancies and AML: A New Role for CD37 in AML. Mol. Cancer Therapeut. *14*, 1650–1660.

Quagliano, A., Gopalakrishnapillai, A., and Barwe, S.P. (2023). Tetraspanins set the stage for bone marrow microenvironment–induced chemoprotection in hematologic malignancies. Blood Adv. *7*, 4403–4413.

Saito, Y., Kitamura, H., Hijikata, A., Tomizawa-Murasawa, M., Tanaka, S., Takagi, S., Uchida, N., Suzuki, N., Sone, A., Najima, Y., et al. (2010). Identification of Therapeutic Targets for Quiescent, Chemotherapy-Resistant Human Leukemia Stem Cells. Sci. Transl. Med. *2*, 17ra9.

Scarfò, I., Ormhøj, M., Frigault, M.J., Castano, A.P., Lorrey, S., Bouffard, A.A., and Scoyk, A.v. (2018). Anti-CD37 chimeric antigen receptor T cells are active against B- and T-cell lymphomas. Blood *132*, 1495–1506.

Sykes, D.B., Kfoury, Y.S., Mercier, F.E., Wawer, M.J., Law, J.M., Haynes, M.K., Lewis, T.A., Schajnovitz, A., Jain, E., Lee, D., et al. (2016). Inhibition of Dihydroorotate Dehydrogenase Overcomes Differentiation Blockade in Acute Myeloid Leukemia. Cell *167*, 171–186.e15.

Vetrie, D., Helgason, G.V., and Copland, M. (2020). The leukaemia stem cell: similarities, differences and clinical prospects in CML and AML. Nat. Rev. Cancer *20*, 158–173.

Wang, Y., Krivtsov, A.V., Sinha, A.U., North, T.E., Goessling, W., Feng, Z., Zon, L.I., and Armstrong, S.A. (2010). The Wnt/β-Catenin Pathway Is Required for the Development of Leukemia Stem Cells in AML. Science *327*, 1650–1653.

Wee, J.L., Schulze, K.E., Jones, E.L., Yeung, L., Cheng, Q., Pereira, C.F., Costin, A., Ramm, G., van Spriel, A.B., Hickey, M.J., and Wright, M.D. (2015). Tetraspanin CD37 Regulates β2 Integrin–Mediated Adhesion and Migration in Neutrophils. J. Immunol. *195*, 5770–5779.

Yan, X., Zhou, Q., Zhu, H., Liu, W., Xu, H., Yin, W., Zhao, M., Jiang, X., and Ren, C. (2021). The clinical features, prognostic significance, and immune heterogeneity of CD37 in diffuse gliomas. iScience *24*, 103249.

Stem Cell Reports, Volume *20*

Supplemental Information

CD37 regulates the self-renewal of leukemic stem cells via integrin-mediated signaling in acute myeloid leukemia

Jinyuan Lu, Lixin Lv, Xiaoxue Tian, Zheng Li, Yuting Ma, Nannan Li, Jian Wang, Guangming Wang, Yu Zeng, Wenjun Zhang, Jun Xu, and Aibin Liang

Supplemental Information

Supplemental Figures

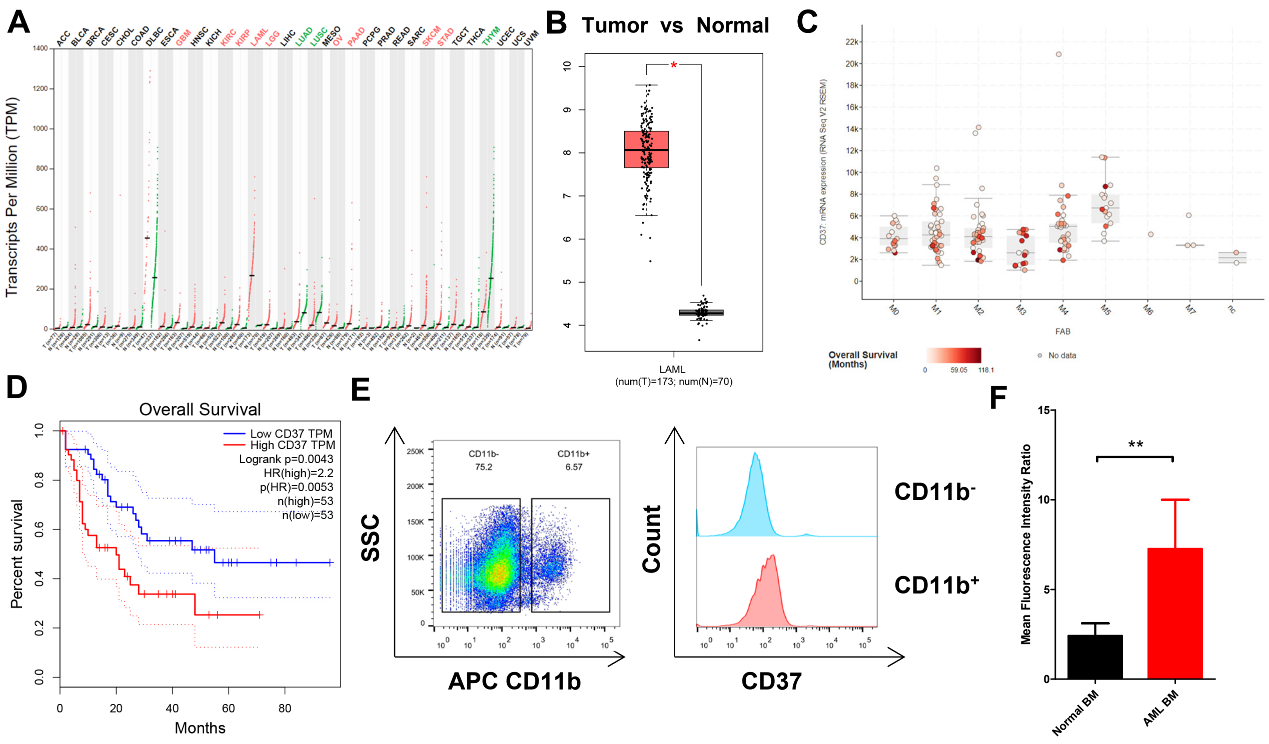

Figure S1. CD37 was upregulated in human AML cells

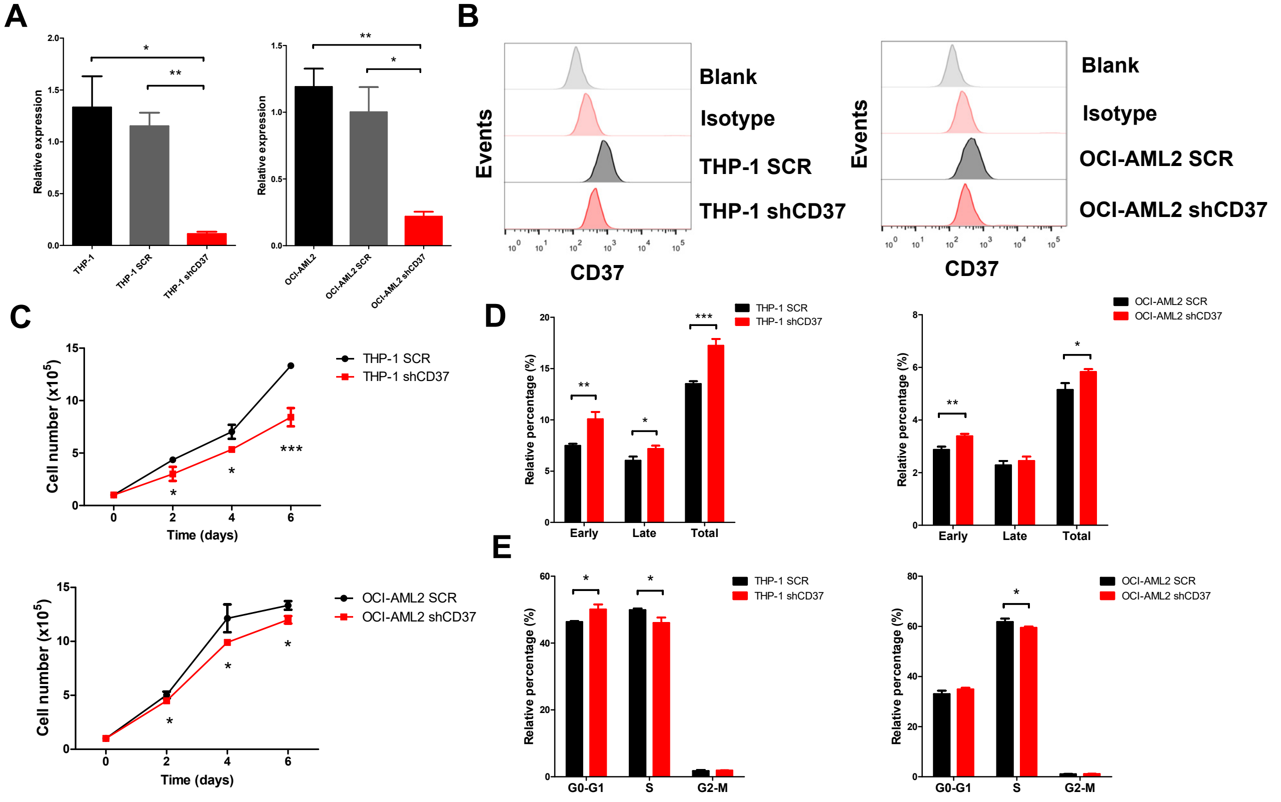

Figure S2. CD37 knockdown inhibited the proliferation of human AML cell lines

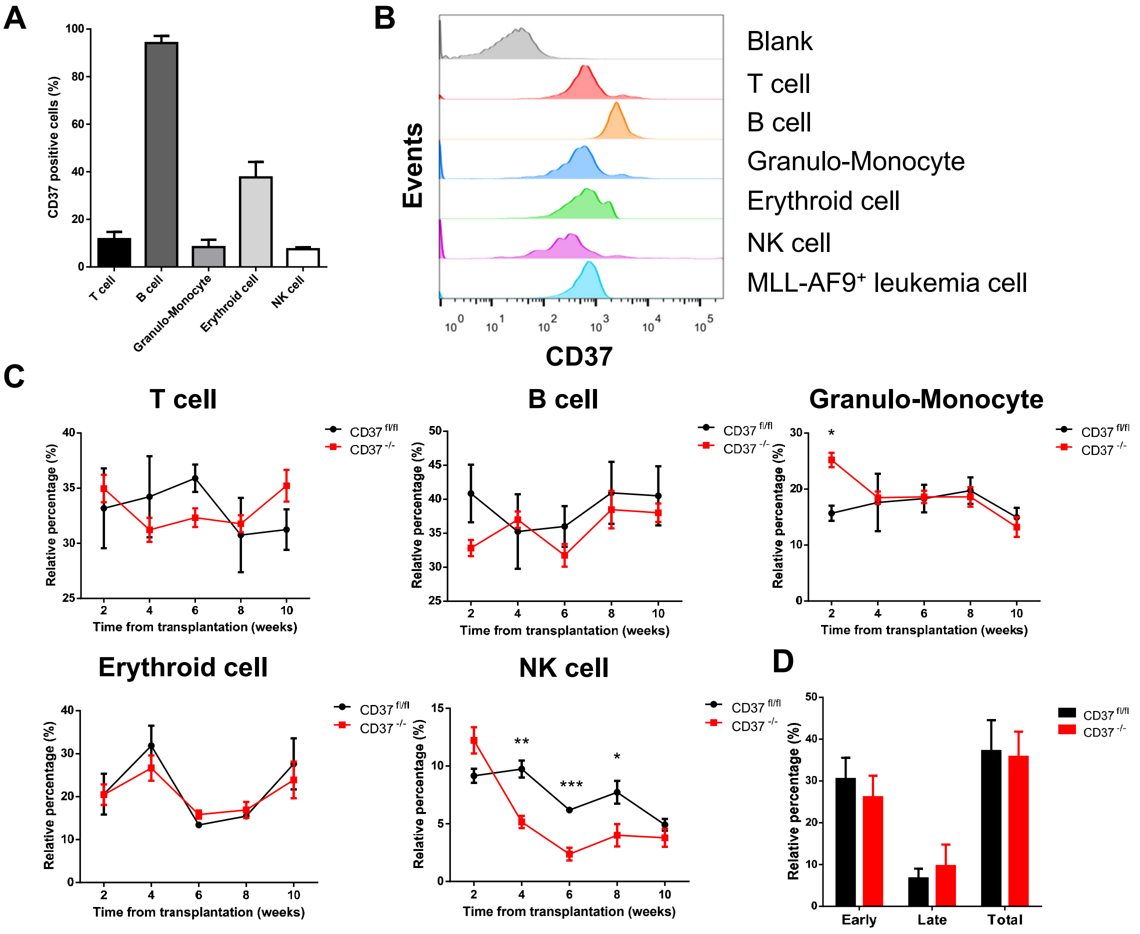

Figure S3. CD37 deficiency had a minor effect on normal BM cell differentiation

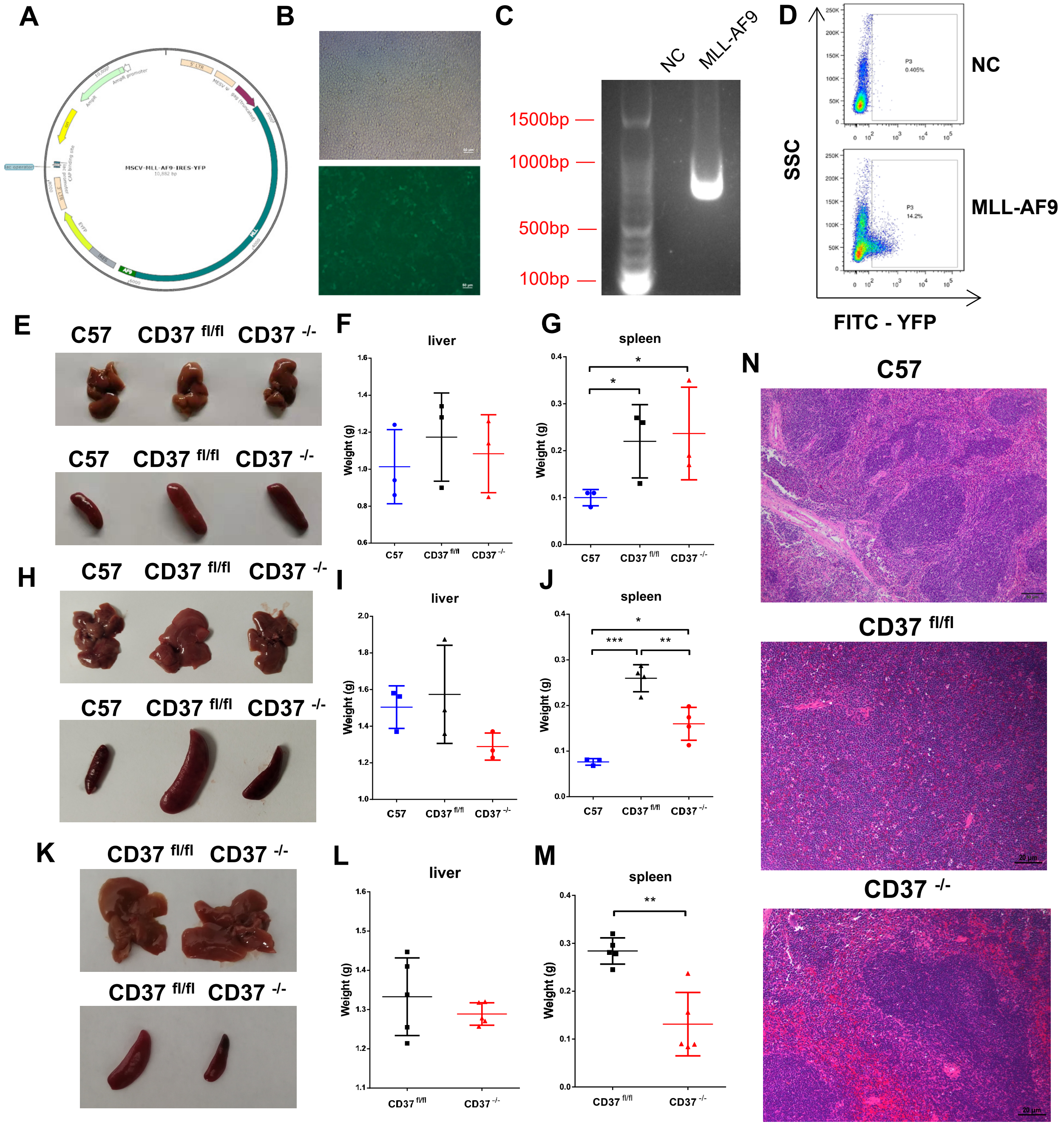

Figure S4. MLL-AF9 transfection efficacy and pathological changes in MLL-AF9 AML

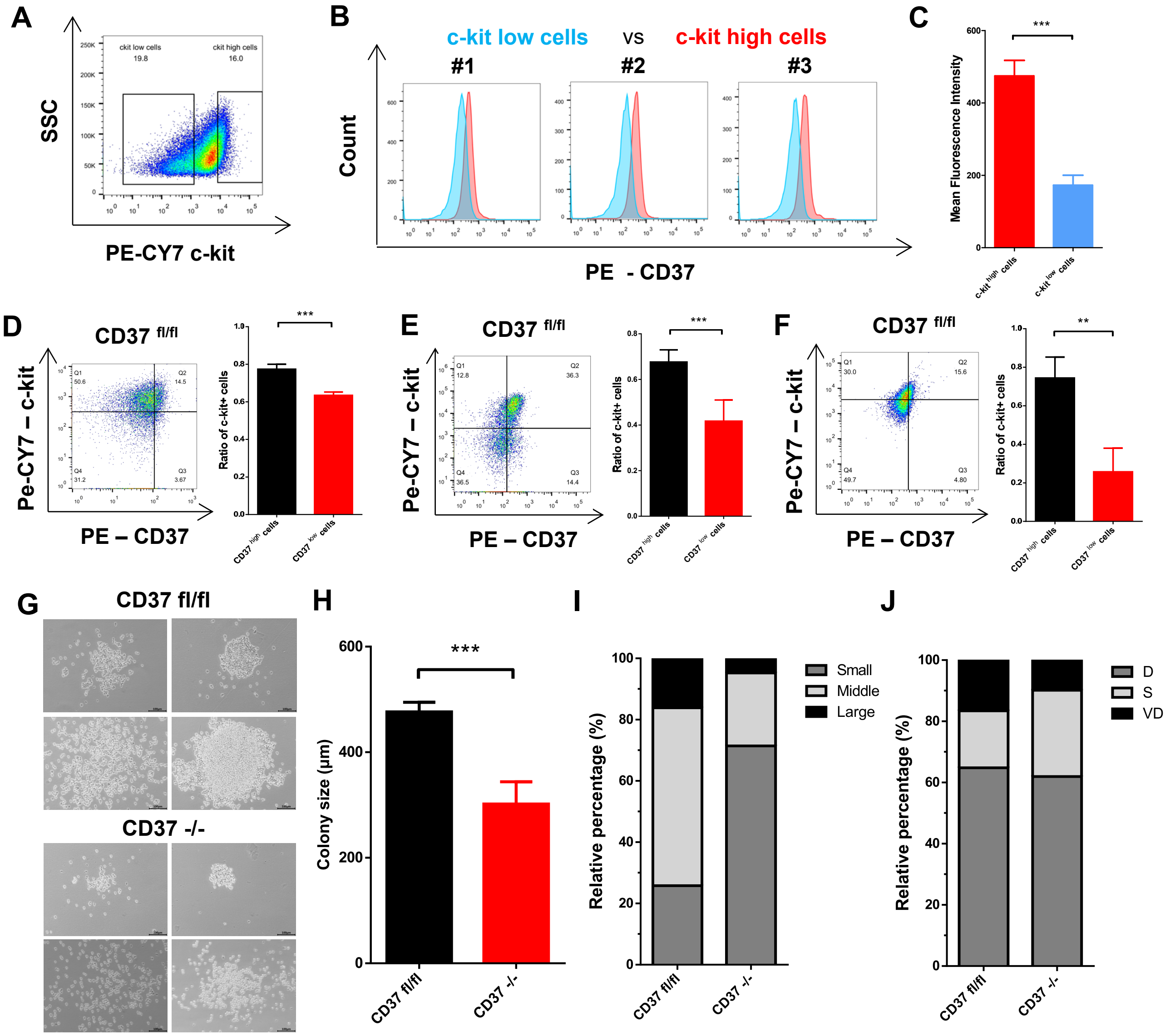

Figure S5. CD37 was enriched in LSCs and CD37 deficiency impaired the colony formation of LSCs

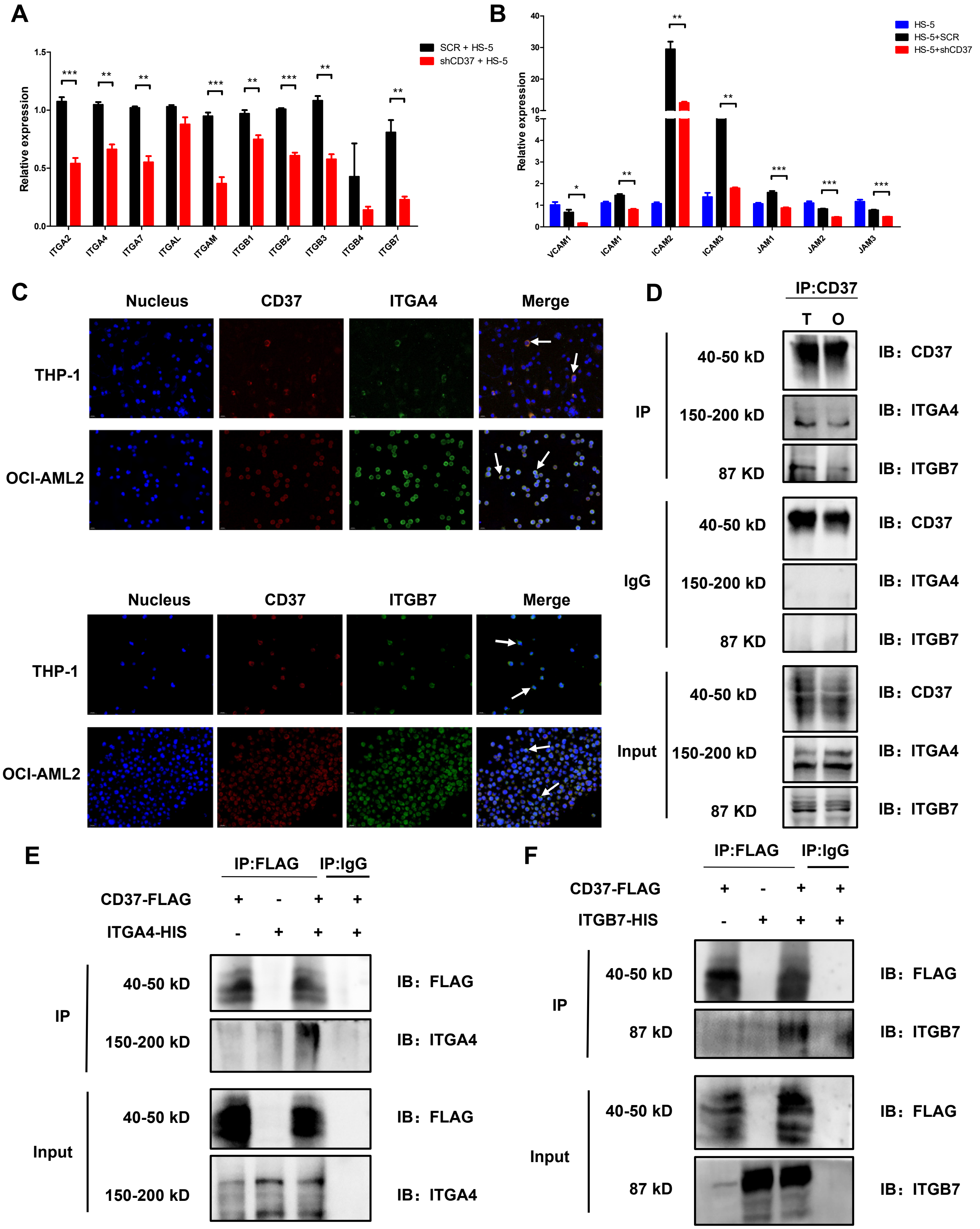

Figure S6. CD37 regulated the expression of integrins and interacted with integrin α4β7

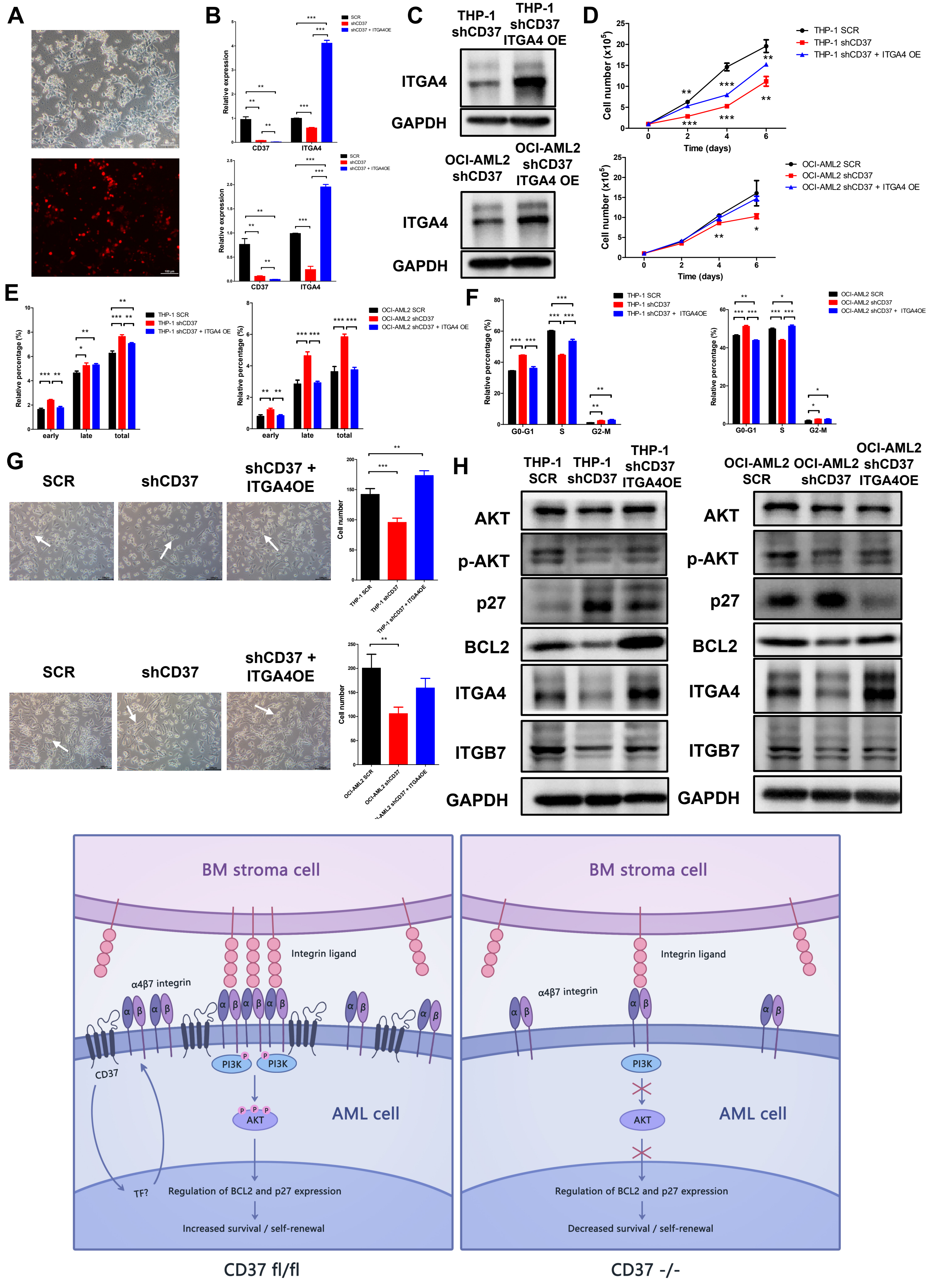

Figure S7. ITGA4 overexpression rescued the phenotypic effects caused by CD37 loss

Supplemental figure legends

Figure S1. CD37 was upregulated in human AML cells.

(A-B) CD37 expression profile among all TCGA tumors (A) or specifically in LAML (B). The data were analyzed using the online database GEPIA.

(C) CD37 expression in different AML subtypes. Dot color represents the duration of overall survival (OS), A deeper red color suggests a longer OS, whereas a lighter white color indicates a shorter OS. The image was depicted on cBioPortal.

(D) Survival plot for LAML patients with high or low expression of CD37. The survival data were analyzed using the online database GEPIA.

(E) Representative flow cytometric analysis of CD37 expression in $CD11b^-$ or $CD11b^+$ AML cells.

(F) The mean fluorescence intensity ratio (MFIR) of CD37 in $CD11b^+$ normal and AML BM cells (n=4 for normal BM and n=7 for AML BM). Error bars in (F) were defined as mean ± SD. $*p < 0.05$, $**p < 0.01$.

Figure S2. CD37 knockdown inhibited the proliferation of human AML cell lines.

(A-B) qPCR and flow cytometric validation of CD37 knockdown efficiency in THP-1 and OCI-AML2 cells with another sequence targeting CD37 (shCD37-2).

(C) Cell proliferation in THP-1 and OCI-AML2 cells transfected with SCR or shCD37.

(D) Quantification of early, late and total apoptotic cells in THP-1 and OCI-AML2 cells transfected with SCR or shCD37.

(E) Quantification of cells in G0-G1 phase, S phase or G2-M phase in THP-1 and OCI-AML2 cells transfected with SCR or shCD37. Error bars in (A), (C), (D) and (E) were defined as mean ± SD. *$p < 0.05$, **$p < 0.01$, ***$p < 0.001$.

Figure S3. CD37 deficiency had a minor effect on normal BM cell differentiation.

(A) CD37 positive rate in murine T cells, B cells, granulo-monocytes, erythroid cells and NK cells (n=4).

(B) Flow cytometric validation of CD37 expression in different types of murine cells.

(C) The trends in the proportions of T cells, B cells, granulo-monocytes, erythroid cells and NK cells in the peripheral blood following tamoxifen or corn oil administration (n=4 for each).

(D) Quantification of apoptotic NK cells in the peripheral blood of CD37 $^{fl/fl}$ and CD37 $^{-/-}$ mice at week 10 (n=4 for each). Error bars in (A), (C) and (D) were defined as mean ± SD. *$p < 0.05$, **$p < 0.01$, ***$p < 0.001$.

Figure S4. MLL-AF9 transfection efficacy and pathological changes in MLL-AF9 AML.

(A) The structure of MSCV-MLL-AF9-IRES-YFP plasmid.

(B) Fluorescence imaging of YFP^+ cells (scale bar = 50μm).

(C) PCR validation of MLL-AF9 expression in Lin^- cells transfected with MSCV-MLL-AF9-IRES-YFP. The PCR primers targeting MLL-AF9 were listed in Supplementary Table 2.

(D) Flow cytometric validation of YFP expression in Lin^- cells transfected with MSCV-MLL-AF9-IRES-YFP.

(E, H and K) The morphology of livers and spleens obtained from normal C57 mice or C57 mice transplanted with $CD37^{fl/fl}$ or $CD37^{-/-}$ AML LSCs. (E) primary AML; (H) secondary AML; (K) tertiary AML.

(F-G, I-J and L-M) Liver and spleen weight of normal C57 mice or C57 mice transplanted with $CD37^{fl/fl}$ or $CD37^{-/-}$ AML LSCs. (F-G) primary AML (n=3); (I-J) secondary AML (n=4); (L-M) tertiary AML (n=5).

(N) Hematoxylin-Eosin (HE) staining for the spleen tissues obtained from normal C57 mice (upper panel, scale bar = 50μm) or C57 mice transplanted with $CD37^{fl/fl}$ or $CD37^{-/-}$ AML LSCs (middle and lower panel, respectively, scale bar = 20μm) in the secondary AML. Error bars in (F), (G), (I), (J), (L) and (M) were defined as mean ± SD. *$p < 0.05$, **$p < 0.01$, ***$p < 0.001$.

Figure S5. CD37 was enriched in LSCs and CD37 deficiency impaired

the colony formation of LSCs.

(A-C) Representative flow cytometric analysis of CD37 expression in c-kit ^{high} or c-kit ^{low} AML LSCs (A-B). The MFI of CD37 in c-kit ^{high} or c-kit ^{low} AML LSCs was also quantified (C, n=3).

(D-F) Representative flow cytometric analysis of CD37 and c-kit expression in CD37$^{fl/fl}$ AML LSCs. The proportion of c-kit^{+} cells in CD37 ^{high} cells or CD37 ^{low} cells was also quantified. (D) primary AML (n=4); (E) secondary AML (n=5); (F) tertiary AML (n=3).

(G) Representative images demonstrating the colony formation of CD37 $^{fl/fl}$ and CD37 $^{-/-}$ AML LSCs in the secondary transplantation model (scale bar = 100μm).

(H) Quantification of colony size for each group (indicated as diameter, μm).

(I) Quantification of small (<370μm), middle (370-630μm) and large (>630μm) colonies for each group. By depicting a histogram to represent colony sizes, optimal intervals for the cutoff values were determined.

(J) Quantification of dense (D), very dense (VD) and sparse (S) colonies for each group. Error bars in (C), (D), (E), (F) and (H) were defined as mean ± SD. $**p < 0.01$, $***p < 0.001$.

Figure S6. CD37 regulated the expression of integrins and interacted with integrin α4β7.

(A-B) qPCR analysis for the expression of integrins on OCI-AML2 cells (A) and integrin ligands on HS-5 stroma cells (B). OCI-AML2 cells transfected with SCR or shCD37 were co-cultured with HS-5 cells for 24 hours and subjected to RNA extraction, cDNA synthesis and qPCR verification.

(C) Representative immunofluorescence images (40x, scale bar = 20μm) demonstrating the location of CD37 and ITGA4/ITGB7 on the membrane of THP-1 and OCI-AML2 cells. The white arrows indicate co-localization of CD37 and ITGA4/ITGB7.

(D) Endogenous co-immunoprecipitation assay for THP-1 (T) and OCI-AML2 (O). CD37 antibody was applied for immunoprecipitation. Simultaneously, rabbit IgG was employed as negative control. A portion of the protein lysates were utilized as input samples. Antibodies targeting CD37, ITGA4 and ITGB7 were administrated for subsequent immunoblotting. The 40-50KD band immunoprecipitated by CD37 antibody in the IgG group represent nonspecific binding of Fc region of the antibody.

(E-F) Exogenous co-immunoprecipitation assay. (E) 293T cells were transfected with CD37-FLAG, ITGA4-HIS or both, and subjected to co-IP and immunoblotting. (F) 293T cells were transfected with CD37-FLAG, ITGB7-HIS or both, and subjected to co-IP and immunoblotting. Error bars in (A) and (B) were defined as mean ± SD. $*p < 0.05$, $**p < 0.01$, $***p <$

0.001.

Figure S7. ITGA4 overexpression rescued the phenotypic effects caused by CD37 loss.

(A-C) Fluorescence imaging, qPCR and Western blot validation of ITGA4 overexpression. (A) Scale bar = 100μm.

(D) Cell proliferation in THP-1 and OCI-AML2 cells transfected with SCR, shCD37 or shCD37 + ITGA4 OE.

(E-F) Quantification for apoptosis (E) and cell cycle distribution (F) in THP-1 and OCI-AML2 cells transfected with SCR, shCD37 or shCD37 + ITGA4 OE.

(G) Representative images demonstrating the adherence of THP-1 cells (upper, scale bar = 100μm) or OCI-AML2 cells (lower, scale bar = 100μm) to HS-5 stroma cells. The white arrows indicate adherent AML cells. The number of adherent AML cells was also quantified.

(H) Immunoblotting for key DEGs in THP-1 and OCI-AML2 cells transfected with SCR, shCD37 or shCD37 + ITGA4 OE.

(I) The schematic diagram illustrating the regulatory role of CD37 in AML. CD37 facilitates the transduction of integrin mediated PI3K-AKT signaling in AML cell lines as well as AML LSCs. In the absence of CD37, integrins (e.g. α4β7) undergo transcriptional downregulation and reduced accumulation, followed by diminished AKT phosphorylation, decreased

BCL2 expression and elevated p27 expression, which collectively contribute to a compromised AML cell survival. Error bars in (B), (D), (E), (F) and (G) were defined as mean ± SD. *$p < 0.05$, **$p < 0.01$, ***$p < 0.001$.

Supplementary Table 1

qPCR primers

**Name**	**Sequence**
muCd37-F	TTTGTCCTTCGTGCCACTG
muCd37-R	AATGAGGATGCCCAGGGTAAT
muCcnd2-F	GAGTGGGAACTGGTAGTGTTG
muCcnd2-R	GCACAGAGCGATGAAGGTC
muCdkn1b-F	TCAAACGTGAGAGTGTCTAACG
muCdkn1b-R	CCGGGCCGAAGAGATTTCTG
muBcl2-F	GCTACCGTCGTGACTTCGC
muBcl2-R	CCCCACCGAACTCAAAGAAGG
muItgb7-F	AAACGGTGCTGCCCTTTGTAA
muItgb7-R	CTCTCTCTCGAAGGCTTGAGC
muPik3cb-F	CTATGGCAGACAACCTTGACAT
muPik3cb-R	CTTCCCGAGGTACTTCCAACT
muGapdh-F	TGACCTCAACTACATGGTCTACA
muGapdh-R	CTTCCCATTCTCGGCCTTG
huCD37-F	CCAACGACTCCACAATCCTAGA
huCD37-R	GGCAAATGCCCACTATGGAAAT
huITGA2-F	CCTACAATGTTGGTCTCCCAGA
huITGA2-R	AGTAACCAGTTGCCTTTTGGATT
huITGA4-F	AGCCCTAATGGAGAACCTTGT

huITGA4-R	CCAGTGGGGAGCTTATTTTCAT
huITGA7-F	CTGACTCCATGTTCGGGATCA
huITGA7-R	CACCTGTGAAGGTTTGGCG
huITGAL-F	TGCTTATCATCATCACGGATGG
huITGAL-R	CTCTCCTTGGTCTGAAAATGCT
huITGAM-F	GCCTTGACCTTATGTCATGGG
huITGAM-R	CCTGTGCTGTAGTCGCACT
huITGB1-F	CAAGAGAGCTGAAGACTATCCCA
huITGB1-R	TGAAGTCCGAAGTAATCCTCCT
huITGB2-F	AAGTGACGCTTTACCTGCGAC
huITGB2-R	AAGCATGGAGTAGGAGAGGTC
huITGB3-F	CATGAAGGATGATCTGTGGAGC
huITGB3-R	AATCCGCAGGTTACTGGTGAG
huITGB4-F	GCTTCACACCTATTTCCCTGTC
huITGB4-R	GACCCAGTCCTCGTCTTCTG
huITGB7-F	GCGCATTGGTTTTGGTTCCT
huITGB7-R	AGATTGCCGGACACACTCTG
huVCAM1-F	GGGAAGATGGTCGTGATCCTT
huVCAM1-R	TCTGGGGTGGTCTCGATTTTA
huICAM1-F	ATGCCCAGACATCTGTGTCC
huICAM1-R	GGGGTCTCTATGCCCAACAA
huICAM2-F	CGGATGAGAAGGTATTCGAGGT

huICAM2-R	CACCCACTTCAGGCTGGTTAC
huICAM3-F	GGAGTTCCTTTTGCGGGTG
huICAM3-R	TCAGAGCTGGGACAATCAGTA
huJAM1-F	GTGCCTACTCGGGCTTTTCTT
huJAM1-R	GTCACCCGGTCCTCATAGGAA
huJAM2-F	GCAGTAGAGTACCAAGAGGCT
huJAM2-R	AGACACTCCGACCCAGTTTCT
huJAM3-F	CGGCTGCCTGACTTCTTCC
huJAM3-R	TGGGGTTCGATTGCTGGATTT
huGAPDH-F	CTGGGCTACACTGAGCACC
huGAPDH-R	AAGTGGTCGTTGAGGGCAATG

Supplementary Table 2

PCR primers

Name	Sequence
CD37-F	GGTTACCCCTGGCTACTGA
CD37-R	ATCCCCCTGTCTCTATCTTGA
Cre-F	GAACCTGATGGACATGTTCAGG
Cre-R	AGTGCGTTCGAACGCTAGAGCCTGT
MLL-AF9-F	CGTCGAGGAAAAGAGTGA
MLL-AF9-R	ATGTTTCCAGGTAACTCTGTAGT
Mycoplasma-F	GGGAGCAAACAGGATTAGATACCCT
Mycoplasma-R	TGCACCATCTGTCACTCTGTTAACCTC

Supplementary Table 3

Human patient information

ID	Gender	Age	Source	Diagnosis
1	Male	68	Bone marrow	AML-M4
2	Female	74	Bone marrow	AML-M1
3	Male	58	Bone marrow	AML-M5
4	Male	34	Bone marrow	AML-M4
5	Female	51	Bone marrow	AML-M5
6	Male	35	Bone marrow	AML-M4
7	Female	75	Bone marrow	AML-M5

Supplemental Methods

Cell lines, animals and patient samples

THP-1, OCI-AML2, MV4-11, K562, NALM-6, JURKAT, HEL, SUP-B15, HL-60, KG-1α, NB4, 293T and OP9 cells were purchased from the Cell Bank of Shanghai Institutes for Biological Sciences. HS-5 was purchased from Shanghai Fuheng Biotechnology Co. Ltd.

CD37 conditional knockout mice ($CD37^{fl/+}$) were established by Shanghai OBiO Technology Co. Ltd using the CRISPR-Cas9 system. *LoxP* alleles were introduced upstream of exon 4 and downstream of exon 5 within the *Cd37* genome. Rosa26-CreERT2 mice were purchased from Nanjing Biomedical Research Institute of Nanjing University. C57 BL/6 J mice (male, 6-8 weeks) were purchased from Shanghai Bikai Keyi Biotechnology Co. Ltd. CD45.1 mice were acquired from Professor Caiwen Duan from Shanghai Jiaotong University School of Medicine as a kind gift. For the generation of $CD37^{fl/fl}$, Cre^+ mice, Mature $CD37^{fl/+}$ mice (F_0) were self-crossed to obtain homozygous $CD37^{fl/fl}$ mice (F_1). Next, $CD37^{fl/fl}$ mice and Rosa26-CreERT2 mice were crossed to obtain $CD37^{fl/fl}$, Cre^+ mice (F_2, F_3, …). The presence of *LoxP* alleles and *Cre* sequences were confirmed by PCR. All mice were kept in specific pathogen-free facilities at the Animal Experiment Center of Tongji University. All animal experiments were conducted in accordance with the ethical approval from

the Animal Ethics Committee of Tongji University.

AML BM samples were obtained from the Hematology Department of Shanghai Tongji Hospital with approval from the Ethical Review Committee of Biomedical Research of Shanghai Tongji Hospital. Informed consent was obtained from all subjects. The clinical information of AML patients was listed in Supplementary Table 3. AML cells were cultured in RPMI 1640 medium with 20% FBS, 1% PS, 10 ng/ml human IL-3, IL-6, SCF, G-CSF and FLT3LG.

ShRNA sequences

The shRNA sequences targeting CD37 was named as shCD37 in this study. A scrambled sequence named SCR was employed as a control sequence to remove off-target effects induced by shRNA. The sequences were as follows:

SCR-F:

GATCCGTGCGCGCTTTGTAGGATTCGTTTCAAGAGAACGAATCC

TACAAAGCGCGCATTTTTG

SCR-R:

AATTCAAAAAATGCGCGCTTTGTAGGATTCGTTCTCTTGAAACG

AATCCTACAAAGCGCGCACG

shCD37-1F:

GATCCGCGACTCCACAATCCTAGATAATTCAAGAGATTATCTAG

GATTGTGGAGTCGTTTTTG

shCD37-1R:

AATTCAAAAACGACTCCACAATCCTAGATAATCTCTTGAATTAT

CTAGGATTGTGGAGTCGCG

shCD37-2F:

GATCCGCTCGATATTCCTGTGCAGAAATTCAAGAGATTTCTGCA

CAGGAATATCGAGTTTTTG

shCD37-2R:

AATTCAAAAACTCGATATTCCTGTGCAGAAATCTCTTGAATTT

CTGCACAGGAATATCGAGCG

Cell apoptosis

Cells were collected and resuspended in 1x Binding Buffer. Annexin V-Alexa Fluor 647 and PI Staining Solution (YEASEN, 40304) were added to the cell suspension, followed by incubation at room temperature for 15min under dark conditions. The cell suspension was then strained with a 70μm filter and subjected to further analysis via flow cytometry. Where indicated, AML cells co-cultured with BM stroma cells were treated with Ara-C (1mM, 1:1000) and incubated at 37°C for 24h. Subsequently, the AML cells were washed 3-4 times with PBS and subjected to cell apoptosis assay.

Cell cycle analysis

Cells were incubated with BrdU (10mg/ml, 1:1000) at 37°C for 2h, and fixed with 75% ethanol at -20°C overnight. The cells were washed with staining buffer (PBS + 1% FBS + 0.09% NaN_3) and resuspended in permeabilization buffer (2N HCl + 0.05% Triton-X100 + ddH_2O) at room temperature for 30min. Subsequently, the samples were washed with staining buffer and resuspended in 0.1M $Na_2B_4O_7$ at room temperature for 2 min. After resuspension in staining buffer, the cells were incubated with APC BrdU antibody (Invitrogen, 17-5071-42) for 30min, and with PI/RNase staining buffer (BD, 550825) for 15min. The cell suspension was then strained with a 70μm filter and subjected to further analysis via flow cytometry.

Cell differentiation assay

The hematopoietic stem cells, progenitors and mature cells were labeled with different antibodies summarized as follows:

LT-HSCs: Lin^-, $c\text{-}kit^+$, $SCA\text{-}1^+$, $FLK2^-$, $CD34^-$; ST-HSCs: Lin^-, $c\text{-}kit^+$, $SCA\text{-}1^+$, $FLK2^-$, $CD34^+$; MPPs: Lin^-, $c\text{-}kit^+$, $SCA\text{-}1^+$, $FLK2^+$, $CD34^+$; CMPs: Lin^-, $c\text{-}kit^+$, $SCA\text{-}1^-$, $CD16/32^{low}$, $CD34^+$, $IL\text{-}7R^-$; MEPs: Lin^-, $c\text{-}kit^+$, $SCA\text{-}1^-$, $CD16/32^-$, $CD34^-$, $IL\text{-}7R^-$; GMPs: Lin^-, $c\text{-}kit^+$, $SCA\text{-}1^-$, $CD16/32^+$, $CD34^+$, $IL\text{-}7R^-$; CLPs: Lin^-, $c\text{-}kit^{low}$, $SCA\text{-}1^{low}$, $IL\text{-}7R^+$; T cells: $CD3^+$; B cells: $CD19^+$; Granulo-Monocytes: $MAC\text{-}1^+$, $GR\text{-}1^+$; Erythroid

cells: TER119$^+$; NK cells: NK1.1$^+$.

Cell adhesion assay

$5x10^5$ YFP$^+$ AML cells were plated on OP9 cells cultured in MEM-α with 20% FBS, 1% PS, 10 ng/ml IL-3, 10 ng/ml IL-6, 10 ng/ml SCF and 10 ng/ml G-CSF in a 12-well plate, and incubated at 37°C for 24h. The wells were washed 3-4 times with PBS in order to remove unattached cells. Images were captured under an inverted microscope and the adherent AML cells were quantified in Image J. For adhesion assay of AML cell lines, $5x10^5$ AML cells were plated on HS-5 cells cultured in DMEM with 10% FBS and 1% PS in a 12-well plate, and the subsequent procedures were consistent with the aforementioned steps.

Transcriptome analysis

$2x10^6$ YFP$^+$, c-kit$^+$ cells isolated from leukemia mice transplanted with CD37$^{fl/fl}$ or CD37$^{-/-}$ AML LSCs were sorted by flow cytometry and lysed with trizol reagent (Invitrogen, 15596026). The samples were sent to Shanghai Hongxu Biotechnology Co. Ltd and sequenced with Illumina Novaseq™ 6000. The downstream analysis was accomplished in R 4.1.1. Differentially expressed genes (DEGs) between CD37$^{fl/fl}$ and CD37$^{-/-}$ AML LSCs were identified with *limma*. *ClusterProfiler* was applied to conduct functional enrichment analysis. The differential genes were further verified

by qPCR and Western blot.

Immunoblotting

Cells were lysed using RIPA lysis buffer (WEIAOBIO, WB0101) supplemented with 1:100 PMSF (Thermo, 36978B) and 1:100 phosphatase inhibitors (YAMEI, GRF102). The lysate was centrifuged and the supernatant was collected and subsequently boiled with 5xSDS protein loading buffer (YAMEI, LT101). Total proteins were separated by SDS-PAGE and transferred onto PVDF membranes (Millipore, IPVH00010), blocked with 5% skim milk dissolved in 1xTBS-T buffer (Solarbio, T1081), and incubated with the corresponding primary antibody at 4°C overnight. Subsequently, the membrane was washed in 1xTBS-T buffer and incubated with the corresponding secondary antibody conjugated with horseradish peroxidase (HRP) at room temperature for 1h. The membrane was then washed in 1xTBS-T buffer, incubated in hypersensitive ECL chemiluminescence reagent (Beyotime, P0018M) and imaged with ChemiScope 6000 (CLINX, 6100).

RNA extraction and quantitative real-time PCR

Total RNA was extracted from cells using Quick-RNA ™ Microprep Kit (ZYMO, R1050 & R1051). The RNA was reversely transcribed into cDNA with FastKing cDNA First-chain Synthesis Kit (TIANGEN, KR116).

Quantitative PCR (qPCR) was conducted on a Roche lightcycler96 Instrument in accordance with the manufacturer's instructions. Relative mRNA expression was quantified and normalized to the reference gene *GAPDH* for each sample. The qPCR primers were listed in Supplementary Table 1.

Immunofluorescence assay

Cells were fixed with 4% paraformaldehyde at 4℃ for 2h and permeabilized with 0.2% Triton X-100 in PBS for 5min. Next, the cells were blocked with 5% BSA at room temperature for 1h and incubated with the corresponding primary antibody at 4℃ overnight. After removal of unbound antibodies with PBS-T buffer, the cells were incubated with the corresponding secondary antibody conjugated with fluorochrome at room temperature for 1h. The cells were then washed with PBS-T buffer and incubated with DAPI for 5min. Finally, the cells were subjected to further observation and imaging under a fluorescence microscope.

Co-immunoprecipitation assay

Cells were lysed using Western and IP Cell Lysis Buffer (Beyotime, P0013) supplemented with 1:100 PMSF and 1:100 phosphatase inhibitors. The lysate was centrifuged and the supernatant was collected, boiled with 5xSDS protein loading buffer (as input) or incubated with anti-CD37

antibody (abcam, ab300400) or rabbit IgG (Beyotime, A7016) at 4℃ overnight (as IP or IgG). The IP and IgG samples were then incubated with protein A+G agarose (Beyotime, P2055) at 4℃ overnight. After removal of unbound antibodies with PBS buffer, the IP and IgG samples were boiled with 1xSDS protein loading buffer and subjected to further Western blot analysis.
